# Dynamics of Antimicrobial Resistance and Genomic Epidemiology of Multidrug-Resistant Salmonella enterica Serovar Indiana ST17 from 2006 to 2017 in China

**DOI:** 10.1128/msystems.00253-22

**Published:** 2022-07-21

**Authors:** Pengcheng Du, Xiaobin Liu, Yue Liu, Ruichao Li, Xin Lu, Shenghui Cui, Yongning Wu, Séamus Fanning, Li Bai

**Affiliations:** a Institute of Infectious Diseases, Beijing Ditan Hospital, Capital Medical University, and Beijing Key Laboratory of Emerging Infectious Diseases, Beijing, People’s Republic of China; b National Health Commission Key Laboratory of Food Safety Risk Assessment, Food Safety Research Unit (2019RU014) of Chinese Academy of Medical Science, China National Center for Food Safety Risk Assessment, Beijing, People’s Republic of China; c Tianjin University of Science & Technology, Tianjin, People’s Republic of China; d Department of Food Science, National Institutes for Food and Drug Control, Beijing, People’s Republic of China; e Shanghai Municipal Center for Disease Control & Prevention, Shanghai, People’s Republic of China; f Jiangsu Co-Innovation Center for Prevention and Control of Important Animal Infectious Diseases and Zoonoses, College of Veterinary Medicine, Yangzhou University, Yangzhou, Jiangsu Province, People’s Republic of China; g State Key Laboratory of Infectious Disease Prevention and Control, National Institute for Communicable Disease Control and Prevention, Chinese Center for Disease Control and Prevention, Beijing, People’s Republic of China; h UCD-Centre for Food Safety, School of Public Health, Physiotherapy and Sports Science, University College Dublin, Dublin, Ireland; UCSF

**Keywords:** *Salmonella enterica* serovar Indiana, antimicrobial resistance, plasmids, population genomics

## Abstract

The genetic features of foodborne Salmonella have changed in recent years as multidrug-resistant (MDR) strains have become prevalent among various serovars. The recent expansion of MDR Salmonella enterica serovar Indiana sequence type 17 (ST17) poses an increasing threat to global public health, as 24.3% (61/251) of *S.* Indiana isolates in this study exhibited resistance to three clinically important antimicrobial agents: fluoroquinolones (ciprofloxacin), extended-spectrum β-lactams (cephalosporin), and macrolides (azithromycin). Both the evolutionary histories and antimicrobial resistance (AMR) profiles of this serovar remain to be described. Bioinformatic analysis revealed multiple lineages have coexisted and spread throughout China. Specifically, emergence of a predominant lineage appears to be associated with accumulated various substitutions in the chromosomal quinolone resistance-determining regions (GyrA S83F D87N and ParC T57S S80R) (141 [56.2%]), as well as acquisition of an extended-spectrum β-lactamase (ESBL)-producing IncHI2 plasmid that has subsequently undergone extensive rearrangement and an IncX1 plasmid that contains *mph*(A), conferring resistance to azithromycin. Several other evolutionary events influencing the trajectory of this drug-resistant serovar were also identified, including sporadic acquisitions of *bla*_CTX-M_-carrying plasmids, along with chromosomal integration of *bla*_CTX-M_ within subclusters. Most human isolates reside in clusters containing isolates from animals, mainly from chickens, indicating the close relationship of human isolates with those from food animals. These data demonstrate that MDR *S*. Indiana ST17 is already widespread and capable of acquiring resistance traits against the clinical important antimicrobial agents, suggesting it should be considered a high-risk global MDR pathogen. The complexity of its evolutionary history has implications for AMR surveillance, epidemiological analysis, and control of emerging clinical lineages.

**IMPORTANCE** The emergence and worldwide spread of AMR Salmonella constitute great public health concerns. S. enterica serovar Indiana is a typical MDR serovar characterized by sporadic reports. However, comprehensive population genomics studies have not been performed on this serovar. This study provides a detailed and comprehensive insight into the rapid evolution of AMR in this important Salmonella serovar in the past 15 years in eight provinces of China. We documented diverse contributory genetic processes, including stable chromosomal integrations of resistance genes, the persistence and evolution of mobile resistance elements within sublineages, and sporadic acquisition of different resistance determinants that occur at all genetic levels (genes, genetic contexts, plasmids, and host strains). There are different mechanisms of antimicrobial resistance in S. enterica serovar Indiana from those of other serovars. This study sheds light on the formation of MDR S. enterica serovar Indiana with chickens as its potential reservoirs and paves the way to curb its further expansion among food animals.

## INTRODUCTION

Nontyphoidal Salmonella enterica (NTS) remains one of the important foodborne pathogens globally (1). It ranks as the most reported bacterial species causing human gastrointestinal infections in many countries (2–4). Moreover, over the past 2 decades, there has been an increasing occurrence of antimicrobial resistance (AMR) associated with NTS (5), especially multidrug-resistant (MDR) phenotypes. These resistant bacteria are more likely to be the causative agents of invasive disease in specific vulnerable populations (6) and outbreaks (7–9). As a result, the World Health Organization (WHO) has deemed antibiotic-resistant S. enterica a critical-priority bacterium, and fluoroquinolone (FQ)-resistant Salmonella spp. were listed by the WHO in 2017 as priority pathogens for which new antimicrobials were urgently needed (10).

Acquisition of AMR genes by foodborne pathogens typically occurs under selective pressure occurring along the food chain (11). In Salmonella, the resistance repertoire varies by serovar (12). Changes in the epidemiology of S. enterica are due to new strains of Salmonella being introduced, interventions (e.g., animal vaccines), or changes in the food chain. Some clonal lineages of MDR Salmonella have shaped the epidemiology of the disease at a global level, as in the case for sequence type 34 (ST34) S. enterica serovar 4,[5],12:i:−, ST313 S. enterica serovar Typhimurium, and ST198 S. enterica serovar Kentucky (9, 13, 14). In China, MDR ST17 S. enterica serovar Indiana has become the most common serovar detected in broilers and is reported among the top three most common serovars from humans in some areas in China (15, 16). Furthermore, certain *S.* Indiana strains are resistant to front-line drugs, including FQs (ciprofloxacin), extended-spectrum β-lactams (cephalosporin), and macrolides (azithromycin [AZM]) approved by the FDA in the United States to treat infections caused by Salmonella (17). Furthermore, certain *S.* Indiana isolates can express resistance to carbapenems or colistin (CT), the last-resort antimicrobials, suggesting that these bacteria have now become a serious challenge for public health (18, 19). The MDR serovar *S.* Indiana has also been reported in many countries recently, from Southeast Asia to North America (20–22), suggesting rapid global emergence and expansion of this zoonotic pathogen across the food chain and an increasing threat to global public health.

Recent studies investigated the genetic basis underpinning resistance to FQs and extended-spectrum β-lactamases (ESBLs) in *S*. Indiana ST17. Mutations in the chromosomal quinolone resistance-determining regions (QRDRs) of DNA gyrase gene (*gyrA*) and DNA topoisomerase IV gene (*parC*) conferred FQs resistance, along with plasmid-mediated quinolone resistance (PMQR) genes [*oqxAB* and *aac(6′)-Ib-cr*]. In addition, diverse *bla*_CTX-M_ genes mapped on mobile genetic elements (MGEs) were reported earlier and found to be integrated into the chromosome (as in the case of *bla*_CTX-M-55_) (15, 23). However, it remains unclear which features of this ST17 clone have resulted in its recent widespread dominance.

Here, we report on the genomic epidemiology and AMR features of 251 *S.* Indiana isolates collected from human and food-related samples in eight provinces of China during 2006 to 2017. Using phenotypic susceptibility data and whole-genome sequencing (WGS) analysis, we determined the prevalence and mechanisms of resistance and identified potential drivers of variation among the AMR profiles described within different lineages.

## RESULTS

### Isolation of *S*. Indiana in China.

Out of the 251 confirmed *S*. Indiana isolates from eight provinces in China, 120 isolates were cultured from clinical samples (118 fecal samples from diarrheal patients and a blood sample and a cerebrospinal fluid sample from two patients with invasive diseases) taken during 2007 to 2017 and 131 isolates from food-related samples taken during 2006 to 2016 (see Table S1 in the supplemental material). All 251 isolates were identified by *in silico* multilocus sequence typing (MLST) as *S*. Indiana ST17 after whole-genome analysis.

10.1128/msystems.00253-22.4 TABLE S1 Source and basic information of Salmonella Indiana isolates. Download Table S1, PDF file, 0.01 MB. Copyright © 2022 Du et al. 2022 Du et al. https://creativecommons.org/licenses/by/4.0/ This content is distributed under the terms of the Creative Commons Attribution 4.0 International license.

### Antimicrobial susceptibility profiles.

**FIG 1:**
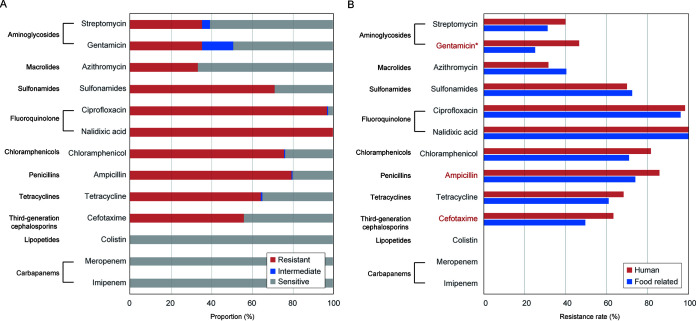
Bar chart showing the prevalence of AMR phenotypes in 251 S. enterica serovar Indiana. (A) Antimicrobial susceptibility of the *S*. Indiana isolates. AMR profiles grouped by the drug class to which *S.* Indiana strains were phenotypically resistant. (B) Resistance rates among the two groups of isolates from human or food-related samples. The resistance rates of the drugs in red are significantly different between the two groups (*P* < 0.05), and for gentamicin the *P* value is <0.001.

Testing of the susceptibility of 251 *S*. Indiana isolates (Table S1) to 13 antimicrobials revealed resistance to 10 of the drugs investigated (Fig. 1A and Table 1), with 217 MDR isolates (87% [217/251]). Among the MDR isolates, more than 72% (156/217) were resistant to ≥6 classes of antimicrobials: 82 isolates were from humans, and 74 were from food-related samples, revealing no significant difference between the two groups (68% versus 56%). All isolates were resistant to nalidixic acid (NAL) and sensitive to imipenem (IPM), meropenem (MEM), and colistin (CT). Approximately 24% (61/251) of the isolates were resistant to all three first-line drugs ciprofloxacin (CIP), cefotaxime (CTX), and azithromycin (AZM). The resistance rates against ciprofloxacin, cefotaxime, and azithromycin were 97.2% (244/251), 56.2% (141/251), and 36.3% (91/251), respectively. For ciprofloxacin, over 85.7% (215/251) of the isolates showed MICs higher than 8 mg/L (Table S2). The resistance rates against gentamicin (GEN) (46.7% versus 25.2%), ampicillin (AMP) (85.8% versus 74.0%), and cefotaxime (63.3% versus 49.6%) were significantly higher in human isolates than food-related isolates (*P* < 0.05) (Fig. 1B). Furthermore, the prevalence of cefotaxime resistance among the isolates from ≤1-year-old patients (76.6% [36/47]) was higher than that from other patients (55.6% [40/72]) (*P* < 0.05).

**TABLE 1:** Differences in resistance rates between human and food-related S. enterica serovar Indiana isolates

Antimicrobial	No. (%) of resistant isolates			*P* value^*a*^
	Total (*n *=* *251)	Human (*n *=* *120)	Food related (*n *=* *131)	
Ampicillin	200 (79.1)	103 (85.8)	97 (74.0)	<0.05
Cefotaxime	141 (55.5)	76 (63.3)	65 (49.6)	<0.05
Imipenem	0 (0.0)	0 (0.0)	0 (0.0)	
Meropenem	0 (0.0)	0 (0.0)	0 (0.0)	
Gentamicin	89 (35.0)	56 (46.7)	33 (25.2)	<0.01
Streptomycin	89 (35.0)	48 (40.0)	41 (31.3)	0.18649
Sulfonamides	179 (70.9)	84 (70.0)	95 (72.5)	0.67736
Chloramphenicol	191 (75.20)	98 (81.7)	93 (71.0)	0.05462
Azithromycin	91 (36.2)	38 (31.7)	53 (40.5)	0.15153
Tetracycline	162 (64.2)	82 (68.3)	80 (61.6)	0.23786
Nalidixic acid	251 (100)	120 (100)	131 (100)	
Ciprofloxacin	244 (97.2)	118 (98.3)	126 (96.2)	0.44972
Colistin	0 (0.0)	0 (0.0)	0 (0.0)	

a *P* values were calculated by chi-square analysis with SPSS version 17.0.

10.1128/msystems.00253-22.5 TABLE S2 MIC value related to the PMQR gene and substitution mutations in QRDR of 251 *S*. Indiana isolates against ciprofloxacin. Download Table S2, DOCX file, 0.02 MB. Copyright © 2022 Du et al. 2022 Du et al. https://creativecommons.org/licenses/by/4.0/ This content is distributed under the terms of the Creative Commons Attribution 4.0 International license.

### Genetic determinants of antimicrobial resistance and genetic elements associated with MDR.

**FIG 2:**
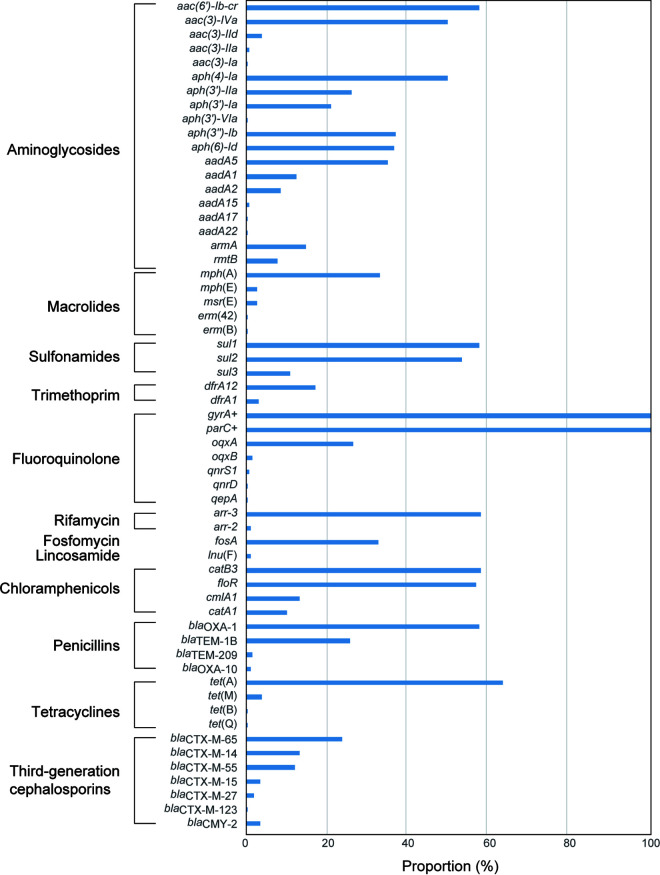
Bar chart showing the prevalence of AMR-associated gene content in 251 *S.* Indiana isolates. Genes detected in the genomes associated with AMR are shown to the left of the graph and are grouped by drug class. Genes that contain point mutations that result in AMR and that are not acquired through horizontal gene transfer are indicated with a cross.

The genomes of 251 isolates were screened for known genetic determinants of AMR, including mobile resistance genes and mutations within QRDRs (Fig. 2 and Table 2; Table S3). For mutations encoding resistance to the clinically important FQs, all isolates had point mutations in QRDRs and possessed amino acid substitutions in GyrA and ParC, with 9 (3.6%), 4 (1.6%), and 238 (94.8%) isolates that had 2-, 3- and 4-amino acid substitutions, respectively (Table S2). The MICs of ciprofloxacin among the isolates carrying 2-substitutions (a GyrA substitution combined with a ParC substitution) ranged from ≤0.125 mg/L to 4 mg/L. In contrast, among 238 isolates carrying 4- substitutions (two GyrA substitutions and two ParC substitutions), the MICs of ciprofloxacin were 8 to 256 mg/L (Table S2). Particularly, the MICs of ciprofloxacin in isolates carrying S83F and D87N amino acid substitutions in GyrA were much higher than those measured in isolates carrying S83F and D87G in GyrA. All isolates with ciprofloxacin MICs up to 256 mg/L (*n* = 11) had S83F and D87N amino acid substitutions in GyrA along with two ParC amino acid substitutions. Furthermore, five PMQR genes were detected, including *aac(6′)-Ib-cr* (*n* = 146), *oqxAB* (*n* = 68), *qnrS1* (*n* = 2), *qnrD* (*n* = 1), and *qepA* (*n* = 1). PMQR genes *aac(6′)-Ib-cr* and *oqxAB* coexisted in 54 isolates (36 were of human origin, and 18 were food related) and were only detected with 4 amino acid substitutions in GyrA and ParC, with MICs of ciprofloxacin ranging from 8 to 256 mg/L (Table S2), while *qnrS1* and *oqxAB* coexisted only in two food-related isolates. Of the 97 isolates possessing amino acid substitutions in GyrA (S83F and D87G) and ParC (T57S and S80R), the detection rate (47.5% [57/120]) in human-derived isolates was higher than that identified in food-related isolates (30.5% [40/131]) (*P* < 0.01). However, of the 141 isolates possessing amino acid substitutions in GyrA (S83F and D87N) and ParC (T57S and S80R), the detection rate in human isolates (47.5% [57/120]) was lower than that in food-related isolates (64.1% [84/131]) (*P* < 0.01).

**TABLE 2:** Characteristics of the six S. enterica serovar Indiana lineages

Characteristic	Isolates, no./total (%)					
	Lineage 1 (*n* = 7)	Lineage 2 (*n* = 2)	Lineage 3 (*n* = 4)	Lineage 4 (*n* = 49)	Lineage 5 (*n* = 48)	Lineage 6 (*n* = 141)
Collection yr						
2006–2011	1/78 (1.3)	2/78 (2.6)		16/78 (20.5)	24/78 (30.8)	35/78 (44.9)
2012–2017	6/173 (3.4)		4/173 (2.3)	33/173 (19.4)	24/173 (13.7)	106/173 (61.1)
Origin of isolates						
Human	2/120 (1.7)	0/120 (0)	4/120 (3.3)	28/120 (23.3)	29/120 (24.2)	57/120 (47.5)
Food related	5/131 (3.8)	2/131 (1.5)	0/131 (0)	21/131 (16.0)	19/131 (14.5)	84/131 (64.1)
QRDRs						
GyrA						
S83F		2/6 (33.3)	4/6 (66.7)			
D87G	7/7 (100)					
S83F D87G				49/97 (51.0)	48/97 (49.0)	
S83F D87N						141/141 (100)
ParC						
T57S	7/9 (77.8)	2/9 (22.2)				
T57S S80R			4/242 (1.7)	49/242 (20.2)	48/242 (19.8)	141/242 (58.3)
PMQR						
*aac(6′)-Ib-cr*				28/146 (19.2)	30/146 (20.5)	88/146 (60.3)
*oqxAB*	2/68 (2.9)		2/68 (2.9)	4/68 (5.9)	24/68 (35.3)	36/68 (52.9)
CTX-M						
*bla*_CTX-M-14_		1/33 (3.0)		3/33 (9.1)	6/33 (18.2)	23/33 (69.7)
*bla*_CTX-M-15_					9/9 (100)	
*bla*_CTX-M-27_				2/5 (40)		3/5 (60)
*bla*_CTX-M-55_	1/30 (3.3)			4/30 (13.3)	2/30 (6.7)	23/30 (76.7)
*bla*_CTX-M-65_	3/60 (5)				15/60 (25)	42/60 (70)
*mph* genes						
*mph*(A)		1/84 (1.2)	1/84 (1.2)		8/84 (9.5)	74/84 (88.1)
*mph*(E)						7/7 (100)
Inc types						
IncHI2	4/133 (3.0)	0/133 (0)	4/133 (3.0)	10/133 (7.5)	36/133 (27.1)	79/133 (59.4)
IncHI2A	4/131 (3.1)	0/131 (0)	4/131 (3.1)	10/131 (7.6)	35/131 (26.7)	78/131 (59.5)
IncN	1/73 (1.4)	1/73 (1.4)	1/73 (1.4)	0/73 (0)	15/73 (20.5)	55/73 (75.3)
IncX1	0/65 (0)	0/65 (0)	0/65 (0)	1/65 (1.5)	0/65 (0)	64/65 (98.5)
IncQ1	1/45 (2.2)	0/45 (0)	3/45 (6.7)	5/45 (11.1)	15/45 (33.3)	21/45 (46.7)

10.1128/msystems.00253-22.6 TABLE S3 Comparisons of the resistance genes of human and food-related S. enterica serovar Indiana isolates. Download Table S3, DOCX file, 0.02 MB. Copyright © 2022 Du et al. 2022 Du et al. https://creativecommons.org/licenses/by/4.0/ This content is distributed under the terms of the Creative Commons Attribution 4.0 International license.

Six *bla*_CTX-M_ subtypes were identified among the 138 ESBL-producing isolates (76 were of human origin, and 62 were food related) distributed in different provinces, including *bla*_CTX-M-65_ (*n* = 60; CTX-M-9 group), *bla*_CTX-M-14_ (*n* = 33; CTX-M-9 group), *bla*_CTX-M-55_ (*n* = 30; CTX-M-1 group), *bla*_CTX-M-15_ (*n* = 9; CTX-M-1 group), *bla*_CTX-M-27_ (*n* = 5; CTX-M-9 group), and *bla*_CTX-M-123_ (*n* = 1; a hybrid of the CTX-M-1 and CTX-M-9 groups). The three dominant subtypes *bla*_CTX-M-65/55/14_ were detected in human and food-related isolates, while *bla*_CTX-M-15/123_ was found only in human isolates. Moreover, the prevalence rate of *bla*_CTX-M-65_ among isolates from ≤1-year-old patients (42.6% [20/47]) was significantly higher than that among isolates from other patients (11.1% [8/72]) (*P* < 0.01), which is in line with the phenotypic characteristics of isolates from these two patient groups. In general, most of the *bla*_CTX-M_ variants have been detected in humans and chickens.

For macrolides, the *mph*(A) gene, which is highly associated with azithromycin resistance, had the highest detection rate of 33.5% (*n* = 84; 34 from humans and 50 food related). The *mph*(E) (*n* = 7), *msr*(E) (*n* = 7), *erm*(42) (*n* = 1), and *erm*(B) (*n* = 1) genes were also detected.

Co-occurrence of one or more AMR genes with mobile genetic elements is a common feature in MDR bacteria. These concatenated AMR genes comprised small mobile genetic elements along with ISs, which then formed variable MDR regions with different gene contents and sizes via recombination mediated by insertion sequences (e.g., IS*26*). The pairwise co-occurrence matrix of AMR genes is shown in Fig. S1, with a few clusters of genes frequently detected together in the same genome. The most common gene network comprised *arr-3* (conferring resistance to rifamycin), *catB3* (conferring resistance to chloramphenicols), *aac(6′)-Ib-cr* (conferring resistance to aminoglycosides), and *bla*_OXA-1_ (conferring resistance to penicillins), which co-occurred in 58.2% of genomes (146/251); the combination of *arr-3*, *catB3*, *aac(6′)-Ib-cr*, and *bla*_OXA-1_ occurred with *sul1* (sulfonamides) in 50.6% (127/251), *aac(*3*)-Iva* (aminoglycosides)*/aph(*4*)-Ia* (aminoglycosides)*/tet*(A) (tetracycline) in 45.8% (115/251), *floR* (chloramphenicols) in 43.4% (109/251), and *sul2* (sulfonamides) in 36.7% (92/251) of genomes. These co-occurring genes were formed into resistance regions comprising IS*26* and *tet*(A), *sul1*, *arr-3*, *catB3*, *bla*_OXA-1_, *aac(6′)-Ib-cr*, *aac(*3*)-Iva*, *aph(*4*)-Ia*, and *sul2* located in ps15D023-IncHI2, ps12177-CTX, pIndS104-CTX, and ps11011-CTX (Fig. S2). Furthermore, *aph(3″)-Ib*, *aph(*6*)-Id*, and *aadA5* co-occurred in 19.1% (48/251) of genomes; the combination of *aph(3″)-Ib*, *aph(*6*)-Id*, and *aadA5* occurred with *oqxAB* in 14.7% (37/251), *fosA* in 12.0% (30/251), *bla*_TEM-1_ in 11.5% (29/251), and *bla*_CTX-M-65_ in 9.2% (23/251) of genomes. These were caused by the resistance region IS*26*-*aph(3″)-Ib*-*aph(*6*)-Id*-*sul2*-IS*26* co-occurring with other resistance genes. Salmonella genomic island 1 (SGI1), the widely reported original chromosomal integron containing an antibiotic resistance gene cluster and identified in several S. enterica serovars (24), was absent from all tested *S*. Indiana isolates.

10.1128/msystems.00253-22.1 FIG S1 Clustering analysis based on the coexistence of resistance genes among the 251 *S.* Indiana isolates. The numbers on the heat map and the corresponding degree of redness represent the number of genomes that the two resistance genes coexisted in. Download FIG S1, TIF file, 2.6 MB. Copyright © 2022 Du et al. 2022 Du et al. https://creativecommons.org/licenses/by/4.0/ This content is distributed under the terms of the Creative Commons Attribution 4.0 International license.

10.1128/msystems.00253-22.2 FIG S2 Sequence comparison of IncHI2 plasmids in *S.* Indiana investigated in this study. Areas shaded in light blue indicate homologies between the corresponding genetic loci on each plasmid. Boxes or arrows represent the ORFs (red, resistance genes; green, integrase, recombinase, and transposase genes; purple, transfer associated; blue, plasmid replication associated; gray, other functions). Download FIG S2, TIF file, 0.9 MB. Copyright © 2022 Du et al. 2022 Du et al. https://creativecommons.org/licenses/by/4.0/ This content is distributed under the terms of the Creative Commons Attribution 4.0 International license.

### Genetic contexts of *bla*_CTX-M_.

Among the 138 *bla*_CTX-M_-positive isolates, 90 (65.2%) could be classified by the locations of *bla*_CTX-M_ in the genome from the fragmented short-read assemblies. *bla*_CTX-M-14_ in 14 isolates (42.4% [14/33]) and *bla*_CTX-M-55_ from 9 isolates (30.0% [9/30]) were located on the chromosome. *bla*_CTX-M-15_ from 9 isolates (100% [9/9]) and *bla*_CTX-M-65_ from 58 isolates (96.7% [58/60]) were carried by plasmids. In addition, 76 *bla*_CTX-M_-positive isolates were from humans and 62 were food related, accounting for 63% (76/120) and 47% (62/131) of the two groups, respectively. Although the positive ratio of *bla*_CTX-M_ was significant higher in human isolates (*P* < 0.05), among the six subtypes, only the positive ratio of *bla*_CTX-M-15_ was significant higher in human isolates (7.5% versus 0% in food-related isolates; *P* < 0.05).

**FIG 3:**
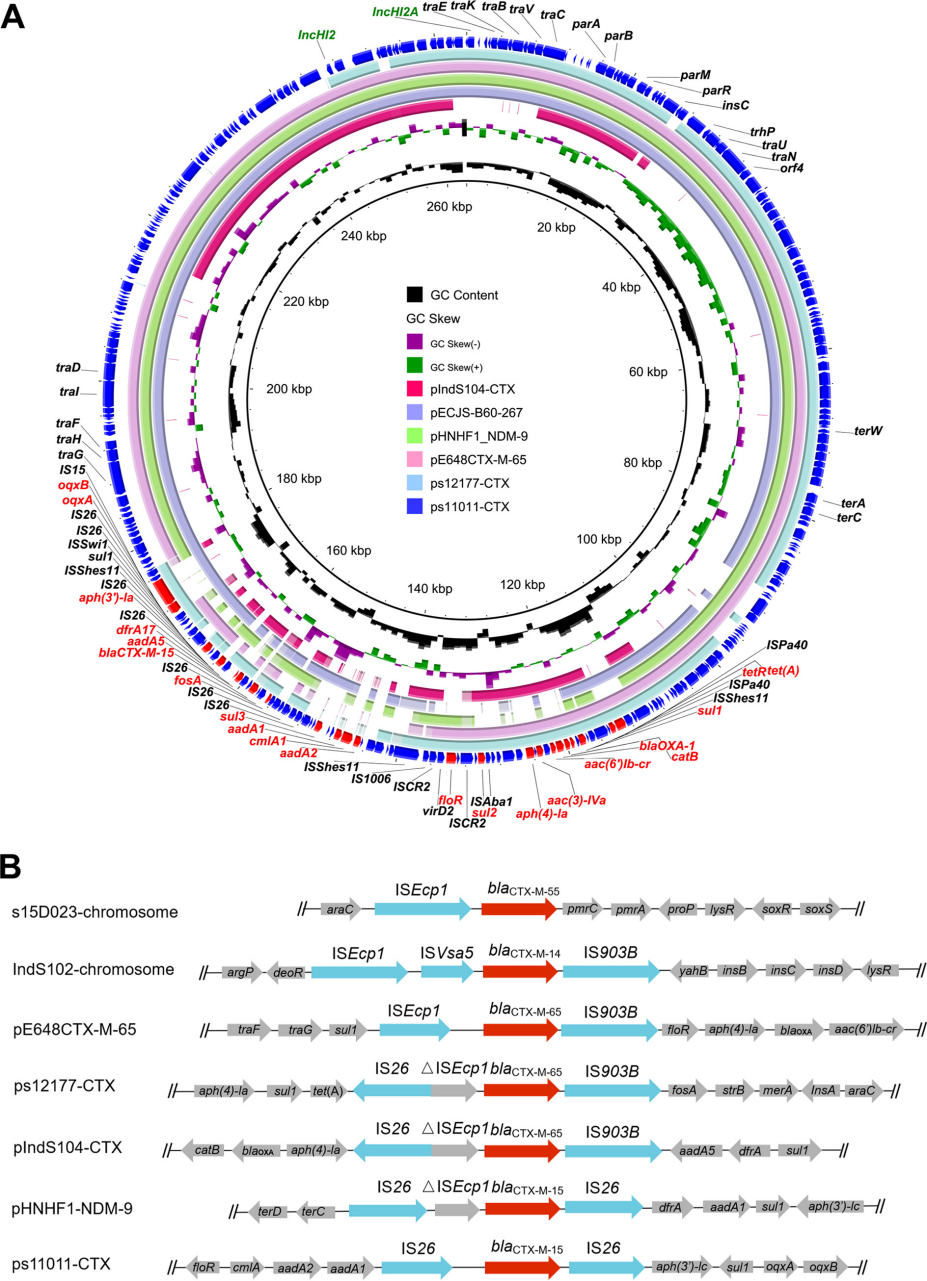
(A) Circular comparison between *bla*_CTX-M_-positive IncHI2 plasmids in this study (pIndS104-CTX, ps17177-CTX, and ps11011-CTX) and other similar IncHI2 plasmids in the NCBI nr database. GC skew and GC content are indicated from the inside out. The arrows represent the positions and transcriptional directions of the ORFs. Genes are differentiated by colors. (B) Genetic environments of *bla*_CTX-M_ in five S. enterica serovar Indiana isolates (s15D023, IndS102, s12177, IndS104, and s11011) with complete genome sequences, along with two other two reported plasmids, pE648CTX-M-65 in E. coli (MN200941.1) and pHNHF1_NDM-9 in K. pneumoniae (CP047668.1). Boxes or arrows represent the ORFs.

To obtain the comprehensive overview of the associated genetic environments of *bla*_CTX-M_ among representative nonclonal isolates from different sources, genome sequences of five isolates (IndS102 from duck, IndS104 from chicken, and s11011, s12177, and s15D023 from human patients) were successfully completed. The IndS102 and s15D023 isolates separately carried *bla*_CTX-M-14_ and *bla*_CTX-M-55_ on their chromosomes within different genetic contexts. Another three isolates carried *bla*_CTX-M_ on IncHI2 plasmids ranging from 200 to 322 kbp and sharing similar core structures (Fig. 3A). Plasmid pIndS104-CTX was the largest *bla*_CTX-M_-bearing IncHI2 plasmid (322,681 bp, 49% GC content) with the specific genetic context IS*26*-IS*Ecp1*-*bla*_CTX-M-65_-IS*903B* located in an MDR region (Fig. 3B). This plasmid shared limited similarity (ca. 100 kbp consisting of an MDR region and a core IncHI2 structure) to other typical IncHI2 plasmids, such as ps11011-CTX, pECJS-B60-267, and pSI85-1 (CP050780; *S*. Indiana). BLASTn analysis demonstrated pIndS104-CTX was most similar (99.99% identity at 100% coverage) to a locus on the chromosome of Chinese *bla*_CTX-M-65_-positve *S*. Indiana SI43, obtained from a spiral shell in China in 2010 (CP050785; ST17), indicating pIndS104-CTX may have the ability to recombine with the chromosome entirely or evolve from ancestor clones like SI43. ps11011-CTX was 263,731 bp in size and possessed 783 predicted coding sequences. ps11011-CTX showed high similarity to ps12177-CTX (64% coverage, 99.98% identity), originating from a human sample from China in 2006, and also to Escherichia coli plasmids pE648CTX-M-65 (MN200941.1; 79% coverage, 99.97% identity) and pECJS-B60-267 (KX254341.1; 78% coverage, 99.95% identity), as well as Klebsiella pneumoniae plasmid pHNHF1_NDM-99 (CP047668.1; 75% coverage, 99.98% identity). ps12177-CTX was 200,106 bp in size (48.61% GC content), and it encoded an MDR region different from that described in ps11011-CTX (Fig. 3). Although *bla*_CTX-M-55_ was located on the chromosome of s15D023, this isolate was positive for a typical IncHI2 plasmid, ps15D023-IncHI2, encoding a shorter MDR region devoid of *bla*_CTX-M-55_. Considering the high prevalence of *bla*_CTX-M_-bearing IncHI2 plasmids, the chromosomal *bla*_CTX-M-55_ may have the ability to incorporate into plasmid ps15D023-IncHI2 via mobile elements and then subsequently transfer to other bacteria by conjugation. The underlying evolutionary trajectory should be further investigated to extend our understanding of these transmission routes of *bla*_CTX-M_ among pathogens.

Although genetic contexts of *bla*_CTX-M_ among 138 isolates could not be resolved completely based on gapped assemblies generated from short-read data, several typical structures were observed. For example, *bla*_CTX-M-15_ was flanked at both ends by IS*26* (Fig. 3B). IS*Ecp1* has been demonstrated to be the most common type of insertion sequence associated with *bla*_CTX-M_, and this was consistent with that in isolates s15D023, s12151, s11066, and IndS102 of this study. In addition, the transposition unit IS*26*-△IS*Ecp1-bla*_CTX-M_-IS*903B* has been identified in several plasmids (e.g., ps12177-CTX and pIndS104-CTX), which might have resulted from the truncation of IS*Ecp1* by IS*26*.

### Genetic contexts of *mph*(A) in the 84 *S.* Indiana isolates.

**FIG 4:**
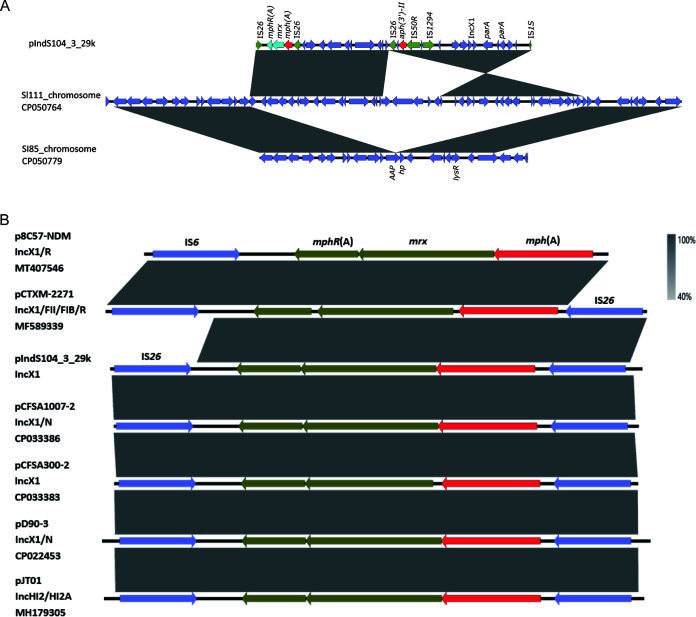
(A) Backbone structures of *mph*(A)-harboring pIndS104_3_29k inserted in the chromosome of Sl111 (CP050764). The conservative region of Sl111 shares high identity with Sl85 (accession no. CP050779) in the NCBI nt database. The regions with >99% homology are indicated by gray shading. (B) Comparison of plasmid pIndS104_3_29k in the present study with different plasmids harboring *mph*(A) from the NCBI database. ORFs with different functions are presented in various colors. ISs are shown with a green arrow. The regions with >99% homology between plasmid pIndS104_3_29k and other *mph*(A)-positive strains are indicated by gray shading. *mph*(A)-positive sequences are aligned in this figure from top to bottom for E. coli (MT407546 and MF589339), S. enterica (CP033386 and CP033383), and *S.* Indiana (CP022453 and MH179305, both from chickens in China), respectively.

Among the five isolates (IndS102, IndS104, s11011, s12177, and s15D023) with complete genome sequences, *mph*(A) was positive in the chicken isolate IndS104 and was located on plasmid pIndS104_3_29k, a typical IncX1 plasmid of size 29,056 bp. It was found to be homologous (>90% coverage, >99.97% nucleotide sequence identity) to six similar plasmids that ranged from 34,764 to 222,492 bp among Salmonella spp. and E. coli, as well as a chromosomal locus in a human *S*. Indiana isolate SI111 (CP050764) (Fig. 4A), which indicates the *mph*(A)-bearing IncX1 plasmids were hypothetically mobilizable and could move into the chromosome via insertion sequences such as IS*26*. The typical IS*26*-*mphR*(A)-*mrx*-*mph*(A)-IS*26* transposition unit was embedded in the IncX1 plasmid and other MDR plasmids such as IncHI2 (Fig. 4B). Linear alignments between plasmid pIndS104-3 and *S*. Indiana SI111 chromosome demonstrated a variant of *mph*(A)-bearing IncX1 plasmid recombined into the SI85 chromosome by IS*26* to generate the chromosomal IncX1 segment in SI111 (Fig. 4A). This highlights the pivotal role of IS*26* in the transmission of *mph*(A) among plasmids and chromosomes. Detailed analysis of *mph*(A)-bearing contigs in the 84 *mph*(A)-positive isolates showed that the core structure *mphR*(A)-*mrx*-*mph*(A) (*n* = 84) and seven additional different core structures were prevalent among these isolates (Fig. S3). However, the complete structures around *mphR*(A)-*mrx*-*mph*(A) were not identified because of short fragmented assembled contigs based on Illumina short-read data.

10.1128/msystems.00253-22.3 FIG S3 Genetic environment comparison of 84 *mph*(A)-harboring *S.* Indiana isolates. Seven different genetic structures are listed above. The numbers of isolates of the seven types are 1, 1, 6, 1, 11, 63, and 1, respectively. Boxes or arrows represent the ORFs. Red arrows represent the *mph*(A) gene. Blue arrows indicate mobile elements. *mphR*(A) and *mrx* are shown by green arrows. Light gray arrows represent the resistance gene and hypothetical protein. A triangle represents the truncated gene. Download FIG S3, TIF file, 1.3 MB. Copyright © 2022 Du et al. 2022 Du et al. https://creativecommons.org/licenses/by/4.0/ This content is distributed under the terms of the Creative Commons Attribution 4.0 International license.

### Phylogenetic analysis and acquisition of AMR determinants during evolution and transmission.

**FIG 5:**
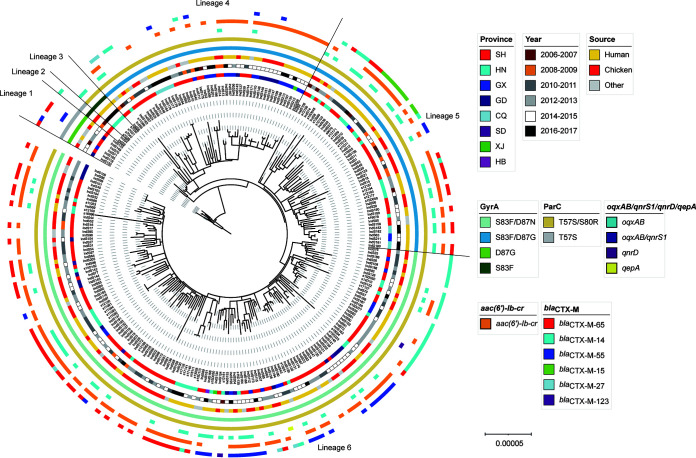
Circular phylogenetic tree of 251 *S.* Indiana isolates reconstructed from SNP data by the maximum likelihood method. From the inner to outer circles, the first circle adjacent to the isolate names shows the different provinces where the strains isolated, the second shows different years when the strains were isolated, the third shows the different sources of strains (human, chicken, or other), the fourth and fifth show quinolone resistance-determining region (QRDR) mutations caused amino acid substitutions in GyrA and ParC, respectively, and the sixth shows the distribution of *bla*_CTX-M_ variants.

Based on the 2,904 core genome single nucleotide polymorphisms (SNPs) obtained from 251 genomes, we performed phylogenic analysis and displayed the phylogenetic relationships of sequenced isolates (Fig. 5). Multiple lineages of *S*. Indiana emerged within the study period and were transmitted to those enrolled provinces. All 251 isolates harbored the mutations on *gyrA* and *parC* associated with the FQ resistance. According to core-genome-based phylogenies, we divided them into six lineages (Fig. 5), which is in line with the continual variation of point mutations in QRDRs of *gyrA* and *ParC* and the corresponding increase of ciprofloxacin resistance. There were three phylogenetically earlier lineages with 2- or 3- amino acid substitutions in GyrA and ParC comprised of minor isolates (13/251 [5.2%]): lineage 1 included seven isolates with D87G in GyrA and T57S in ParC, lineage 2 included two isolates with S83F in GyrA and T57S in ParC, and lineage 3 included four isolates with S83F in GyrA and T57S and S80R in ParC. The other three later lineages with 4- amino acid substitutions were the dominant groups. Lineage 4 (*n* = 49) and lineage 5 (*n* = 48) had S83F and D87G in GyrA and T57S and S80R in ParC, while lineage 6 (*n* = 141) had S83F and D87N in GyrA and T57S and S80R in ParC, which was the most common lineage (141/251 [56.2%]). Moreover, in the early stages of quinolone resistance evolution, most of the isolates with double-amino-acid substitutions in GyrA (D87G [lineage 1] or D87F [lineage 2]) and ParC (T57S) were susceptible to ciprofloxacin, except in two isolates that also carried PMQR genes (*oqxAB* and *qnrS1*), and the isolates from lineage 3 with three amino acid substitutions in GyrA (S83F) and ParC (T57S and S80R) exhibited low MIC values of ciprofloxacin (Fig. 5). In contrast, high-level quinolone-resistant isolates had 4 amino acid substitutions in GyrA and ParC. For GyrA, the resistant isolates carried two amino acid substitutions of S83F and D87G (97 [38.6%]) initially (lineage 4 and lineage 5) and then had additional changes into S83F and D87N (141 [56.3%]) during evolution (lineage 6) (Table 2). Of note, *bla*_CTX-M-15_ was completely restricted to lineage 5. Genotypes *bla*_CTX-M-14_, *bla*_CTX-M-55_, and *bla*_CTX-M-65_ were also mostly discovered within lineage 6, accounting for 69.7% (23/33), 76.7% (23/30), and 70% (42/60) of the isolates harboring each genotype, respectively (Fig. 5 and Table 2). Moreover, the majority of human isolates reside in clusters containing isolates cultured from animal samples, mainly from chickens, indicating the high genomic similarity of human isolates to chicken isolates. Isolates from most geographic provinces were identified within the dominant lineages 4, 5, and 6, although there were smaller, geographically restricted clusters also identified within these. Interestingly, *mph*(A) appeared most frequently in lineage 6 (88.1% [74/84]), followed by lineage 5 (9.5% [8/84]), and only occurred once in lineage 2 and lineage 3, respectively. Furthermore, co-occurrence of *mph*(A) with diverse ESBL genes was observed (*bla*_CTX-M-65_ [*n* = 30], *bla*_CTX-M-15_ [*n* = 7], *bla*_CTX-M-55_ [*n* = 6], *bla*_CMY-2_/*bla*_CTX-M-14_ [*n* = 5], *bla*_CTX-M-14_ [*n* = 4], and *bla*_CTX-M-27_ [*n* = 2]). Both IncN and IncX1 replicons were dominantly distributed in lineage 6, with prevalence of 75.3% (55/73) and 98.5% (64/65). Based on the complete genome sequences of IndS104, *mph*(A) was located in an IncX1 plasmid. In addition, among the 251 isolates, *mph*(A) and IncX1 were both detected in 55 isolates belonging to lineage 6, and both were negative in 157 isolates. The distribution of *mph*(A) and IncX1 displayed a significant correlation with a calculated coefficient of 0.64 (95% confidence interval [CI], 0.56 to 0.71; *P* < 0.05). The isolates carrying IncX1 plasmids might be more likely to carry *mph*(A) (odds ratio, 29.2).

## DISCUSSION

The genetic features of foodborne Salmonella have changed significantly in recent years as MDR Salmonella strains have become prevalent among various serovars, such as *S*. 1,4,[5],12:i:−, *S*. Kentucky, and S. enterica serovar London (25). In China, *S*. Indiana has emerged as the second most common serovar isolated from poultry after S. enterica serovar Enteritidis (15). Moreover, ESBL-producing *S*. Indiana has been detected in the United States in food imported from China (20). As one of the largest global chicken-producing and -consuming countries, we collected 251 *S*. Indiana isolates both from humans and food-related samples in eight Chinese provinces from 2006 to 2017. This is the first nationwide genomic epidemiology study of *S*. Indiana ST17 in China, which highlights the nationwide transmission and localized lineage expansion of this serovar. In addition, the results support both transmission and localized lineage expansion following specific introductions into a geographic locality. There is high genomic similarity between human isolates and chicken isolates, and the latter might function as the source of human infections.

In Salmonella, antimicrobial resistance varies by serovar. Our previous data showed that 85.8% of the study isolates represented an MDR phenotype, which is much higher than those reported for other MDR serovars in China (S. enterica serovar Rissen [76.2%], *S.* Typhimurium [75.7%], and *S.* London [75.0%]) (26). Furthermore, the prevalence of resistance to nalidixic acid (100%) and ciprofloxacin (97.2%) was extremely high in *S.* Indiana. We hypothesize that this high-level resistance is linked to strong selective pressure exerted by FQs use in poultry, *S*. Indiana’s main reservoirs (27). Since 2016, four FQs have been forbidden for use in food-producing animals in China (announcement no. 2292 of the Ministry of Agriculture of the People's Republic of China). The evolution of FQs resistance is usually mediated by the accumulation of multiple mutations in a stepwise process in relation to the host strain’s fitness (28). Our whole-genome sequencing (WGS) analysis established that FQ resistance mutations in *gyrA* and *parC* were lineage associated, as reported within ST131 of *E. coli* (29). Recent studies reported that the strains with S83F and D87N substitutions in GyrA (lineage 6) have a higher MIC value for FQ than those with S83F and D87G (lineage 5) (30). It has been shown that more intense FQ resistance might provide ST131 H30, especially H30Rx, with subtle fitness advantages over other FQ-resistant E. coli strains (29). This might explain why lineage 6, including 141 isolates (56.2%) with a high MIC, became more prominent than others. PMQR genes located in the chromosomes and plasmids are often detectable in ciprofloxacin-resistant Salmonella isolates of various serovars (31). In contrast to *qnrS1* in most other Salmonella serovars, *aac(6′)-Ib-cr* was the dominant genotype, with the carriage rate increasing from 20% in 2006 to 65% in 2017 (31), followed by *oqxAB*. Unlike the most common PMQR gene cluster, the *qnrS1*-*oqxAB* combination identified in 66% (375/566) of the ciprofloxacin-resistant Salmonella strains of various serovars, the *oqxAB* and *aac(6′)-Ib-cr* combination was the predominant one in our study, which was located in the IncHI2 plasmid. This gene combination was also located in a nonconjugative plasmid, pCFSA244-1, mainly transmitted among *S.* Typhimurium isolates, and *qnrS2*-*aac(6′)-lb-cr*-*oqxAB* elements were also found on the chromosome in S. enterica serovar Derby (31). However, the *oqxAB*-*aac(6′)-Ib-cr*-bearing IncHI2 plasmid found in *S*. Indiana was mobilizable by conjugation (32). The high-level FQ resistance was conferred by two GyrA substitutions along with the two ParC substitutions, and the presence of an additional two PMQR genes in *S*. Indiana seriously compromises treatment options, especially for human invasive cases. The significantly higher detection rate of PMQR genes we observed in clinical isolates might be related to the common use of FQs in patients as an extra selective condition during the infection and enrollment of human isolates compared with the food-related isolates. This was also in line with the higher carriage rate of PMQR genes among isolates representing higher MICs of ciprofloxacin. In addition, combinations of S83F and D87G substitutions in GyrA were significantly prevalent in human isolates, but combinations of S83F and D87N substitutions in GyrA were significantly rare, which might be related to the different fitness costs of these mutations in the human body environment and needs further study. The alternative explanation is sampling bias, due to the isolates being from an epidemic surveillance not a well-designed randomized study. We also observed that simultaneous multiple mutations in QRDRs lead to high-level FQ resistance, which was in line with previous studies (33). This might because the simultaneous structural variations on the dual FQ targets of gyrase and topoisomerase IV result in significantly higher impact on the binding and functioning of FQs (34).

In the collection, 56.2% of isolates were resistant to cefotaxime. Different variants of the CTX-M families were detected in *S*. Indiana, contributing to their cefotaxime resistance phenotypes (18). Among the provinces in which we collected isolates, the results showed that *bla*_CTX-M-65_ was the most widely distributed, followed by *bla*_CTX-M-14/55_, contrasting with reports of isolates collected in Vietnam, wherein the detection rate of *bla*_CTX-M-27_ was the highest (35). However, our findings are consistent with previous studies in China reporting that *bla*_CTX-M-65_ and *bla*_CTX-M-14_ were the most common genotypes expressing bacterial resistance to cefotaxime (36, 37). In addition to Asia, the emerging *bla*_CTX-M-65_-producing S. enterica serovar Infantis was isolated from patients and retail chicken meat in the United States (38), and the AMR genes located on the IncFIB-like megaplasmid pESI (plasmid for emerging *S*. Infantis-like) (39). Furthermore, *bla*_CTX-M-15_ was only found in *S.* Indiana from humans in this study, which illustrates different food vehicles of the human isolates or different sources of *bla*_CTX-M-15_-harboring genetic elements. In contrast, the one substitution (A80V) variant of *bla*_CTX-M-15_, *bla*_CTX-M-55_, possessing enhanced cephalosporin-hydrolyzing activity, was both prevalent in both isolates from humans and those from food-related samples, particularly in lineage 6, indicating potential higher adaptability of *bla*_CTX-M-55_. IncHI2 plasmids were typical MDR plasmids positive for *bla*_CTX-M_, *mcr-1*, class 1 integrons, and *oqxAB*, similar to those found in other *Enterobacteriaceae* (Salmonella spp. and E. coli) (40, 41). Furthermore, the *bla*_CTX-M_-harboring conjugative IncHI2 plasmids, which act as the transmission facilitator of *bla*_CTX-M_ between Salmonella and other species, could be the origin of chromosomal *bla*_CTX-M_ in Salmonella serovars. The core mobile elements around *bla*_CTX-M_ might play important roles during its transmission between chromosomes and plasmids. This was manifested by both IS*Ecp1*-*bla*_CTX-M-55_ and IS*Ecp1*-IS*Vsa5*-*bla*_CTX-M-14_-IS*903B* translocated into chromosomal core regions in s15D023 and IndS102, respectively. Undoubtedly, chromosomal *bla*_CTX-M_ could stabilize during cell reproduction, which may be the evolutionary destiny of *bla*_CTX-M_ in Salmonella serovars such as *S*. Indiana in this study. However, for the genetic contexts of *bla*_CTX-M-65_ in *S*. Indiana, most were found on IncHI2 plasmids (32). The *bla*_CTX-M-55_ in our study is also one of the critical genes that can express resistance, which shows that the prevalence of *bla*_CTX-M-55_ in China has increased, thereby exacerbating the resistance to cephalosporins (42). The occurrence of *bla*_CTX-M-55_-producing Salmonella increased significantly from 5.9% in 2010 to 2011 to 23.5% in 2016 to 2017 (*P* < 0.05). Most *bla*_CTX-M-55_ genes in *S*. Indiana are located on the chromosome, which is different from its plasmid origin in other serovars, such as S. enterica serovars Enteritidis, Goldcoast, Typhimurium, and Choleraesuis (43–46).

Macrolide (azithromycin) resistance was determined in 36.3% (91/251) of *S.* Indiana isolates harboring the plasmid-borne *mph*(A), *mph*(E), *erm*(42), and *erm*(B) genes. Moreover, in the present study, 24% of isolates were coresistant to azithromycin, ciprofloxacin, and cefotaxime, which is considerably higher than the levels reported in a Chinese national surveillance study (0.2%) from 2005 to 2011 (47) and a Chinese regional study (1.7%) from 2014 to 2017 (26). In the United States, though the trend of association of national NTS isolates with azithromycin resistance remains low (3.7 per 1,000 in 2015 and 2016), the rise is associated with the emergence of plasmid-mediated macrolide resistance genes *mph*(A) and *mph*(E), raising concern about the spread of resistance among bacteria (48). In our study, *mph*(A)-positive isolates were mostly grouped into lineages 5 and 6 coexisting with diverse ESBL genes. This feature is highly related to the IncX plasmid, which has played an important role in the spreading of resistance genes, such as *bla*_NDM_ (IncX3) and *mcr-1* (IncX4 and IncX2) (49–51). Moreover, resistance determinants may enter other countries via international travelers (48).

It has been reported that plasmids with IncA/C, B/O, HI1, HI2, I1, N, F, and P replicons are often associated with MDR in Salmonella. The high prevalence of IncHI2 plasmids in *S*. Indiana is similar to that reported for *S.* Typhimurium (52). Meanwhile, AMR genes can accumulate in the same plasmid, such as IncHI2 like a nested Russian doll (53), and co-occur on plasmids (IncX1) carrying other genes encoding resistance to the highest-priority “critically important antimicrobials,” which challenges public health. In this study, we also observed some differences between antimicrobial susceptibility phenotypes and the corresponding AMR genes among phylogenetic lineages and isolates from different sources, like clinical isolates and food-related isolates. These results were in line with many pathogens widely distributed in human and food animals. The resistance and AMR gene profiles were more varied in isolates from food animals than clinical isolates, which might be caused by the higher diversity of food animals and the breeding environments. There would be selections of different isolates during the transmission via the food chain, and adaptive changes would also occur when the pathogens infect humans (54).

### Conclusion.

This study has provided a detailed and comprehensive insight into the rapid evolution of MDR in *S.* Indiana in the past 15 years in China. There are different mechanisms of antimicrobial resistance in *S*. Indiana compared to other serovars. We documented diverse contributory genetic processes, including stable chromosomal integrations of resistance genes, persistence and evolution of mobile resistance elements within lineages, and sporadic acquisition of different resistance determinants. This might be linked to a diverse host niche, including several animal reservoirs, indicating the necessity for a One Health approach to monitor the spread and source of resistance efficiently. The diversity of resistance profiles within *S*. Indiana also calls for further control supported by continuous surveillance strategies that target both bacterial strains and their mobile genetic elements.

## MATERIALS AND METHODS

### Collection of *S*. Indiana isolates.

A total of 251 confirmed *S*. Indiana isolates were collected and analyzed. Clinically associated isolates originated from samples collected previously by the State Key Laboratory of Infectious Disease Prevention and Control from five provinces in China during 2007 to 2017. The other isolates were cultured from food-related samples, including animals (chickens, ducks, frogs, fish, and shells) and food (egg, pork, bean, and dairy products) collected previously by the National Health Commission Key Laboratory of Food Safety Risk Assessment from four provinces in China during 2006 to 2016. In total, isolates from eight provinces were included. Clinical fecal samples were enriched in selenite brilliant green sulfa enrichment broth for 18 h at 37 ± 1°C, and anatomical site samples (e.g., blood or cerebrospinal fluid) were enriched on blood agar plates for 18 h at 37 ± 1°C. Clinical sample isolates were purified as described previously (55). S. enterica isolates from food and environmental samples were isolated using a modified method based on the United States Department of Agriculture Food Safety and Inspection Service *Microbiology Laboratory Guidebook* (56). The isolates with typical Salmonella phenotypes from clinical samples and food-related samples were all identified on the Vitek 2 Compact automated microbial identification platform (bioMérieux, Beijing, China), along with amplification of the *invA* gene by PCR. All isolates were identified to the serogroup level and then serotyped by slide agglutination with commercial Salmonella antisera (Statens Serum Institute, Denmark) following the Kauffmann-White scheme at the National Health Commission Key Laboratory of Food Safety Risk Assessment in Beijing, China.

### Antimicrobial susceptibility testing.

Antimicrobial susceptibility testing (AST) of the *S*. Indiana isolates was performed by the agar dilution method and interpreted according to 2018 Clinical and Laboratory Standards Institute (CLSI) guidelines (57) and the European Committee on Antimicrobial Susceptibility Testing (EUCAST; https://eucast.org/). The antimicrobial susceptibilities of the following antimicrobials were assessed: ampicillin (AMP), cefotaxime (CTX), cefotaxime-clavulanic acid (CTX-CLA), chloramphenicol (CHL), ciprofloxacin (CIP), nalidixic acid (NAL), gentamicin (GEN), streptomycin (STR), imipenem (IPM), meropenem (MEM), tetracycline (TET), azithromycin (AZM), sulfonamide (SUL), and colistin (CT). The MICs were calculated. Multidrug resistance was defined as resistance to three or more classes of antimicrobials. Escherichia coli ATCC 25922 and Klebsiella pneumoniae ATCC 700603 were used as the quality control strains.

### Whole-genome sequencing and detection of AMR genotypes.

Genomic DNA was extracted using the Wizard Genomic DNA purification kit (Promega, Madison WI) and then sequenced using an Illumina HiSeq 2500 platform (Illumina, San Diego, CA) to generate 150-bp paired-end reads from a library with an average insert size of 500 bp. Raw reads were filtered to remove low-quality reads by fastQC (58) and then assembled using SPAdes v3.13 with the default parameters (59). The assembled genomes were evaluated by Quast (60). Prokka was used to perform the gene prediction and annotation (61), and then bioinformatic tools, including Resfinder, ISFinder, and PlasmidFinder, were used to analyze antimicrobial resistance genes (ARGs), insertion sequences (ISs), and plasmid incompatibility (Inc) types (62–64). The ARGs were identified based on the best alignment with the ResFinder database, with thresholds of 90% DNA sequence identity and minimum coverage of 80%. An *in silico* multilocus sequence typing (MLST) scheme was used to subtype the isolates using BLASTn and seven housekeeping genes: *aroC*, *dnaN*, *hemD*, *hisD*, *purE*, *sucA*, and *thrA* (65).

### Analysis of *bla*_CTX-M_ genomic locations.

Locations of *bla*_CTX-M_ were determined after whole-genome sequence analysis. *bla*_CTX-M_-containing contigs were examined for plasmid Inc types using PlasmidFinder (62). Based on the draft genome analysis, the *bla*_CTX-M_-carrying contigs were categorized and clustered initially. Five representative isolates harboring differed *bla*_CTX-M_-carrying contigs were selected for complete genome construction using long-read sequencing on the MinION long-read sequencing platform. The genomes were assembled via *de novo* hybrid assembly using Unicycler v0.4.4 (66, 67). The draft genomes of the other isolates were compared with *bla*_CTX-M_-carrying chromosomes or plasmids in the five representative complete genomes using BLASTn to validate whether the *bla*_CTX-M_-carrying contigs were on chromosomes or plasmids (68), with thresholds of 95% DNA sequence identity and minimum coverage of 80% of the contigs (69).

### Phylogenetic analysis.

The complete genome sequence of *S.* Indiana D90 (accession no. CP022450) was used as the reference in phylogenetic analysis. Illumina reads were mapped to the reference genome using Bowtie 2 v2.2.8, with single nucleotide polymorphisms (SNPs) identified using Samtools v1.9, and the data were combined as described previously (24, 70). The high-quality SNPs (hqSNPs) supported by more than 5 reads with a mapping quality of >20 were investigated further (https://github.com/generality/iSNV-calling). Multiple alignments of core genomes identified from the pairwise alignments with *S.* Indiana D90 were used as the input for Gubbins to detect and remove recombination sites (71). Phylogenetic analysis was done based on the remaining core genome sequences. The best-fitting substitution model (K3P+ASC+R2) was identified using ModelFinder (72) and selected to build a maximum likelihood phylogenetic tree using IQ-TREE v 2.0.6 (73). The consensus tree was constructed from 1,000 bootstrap trees using UFBoot2 (74). The phylogenetic relationship and distribution of resistance genes were displayed using iTOL (75).

### Data availability.

The genome sequences in this study were deposited into the National Center for Biotechnology Information database under BioProject accession no. PRJNA850394.
