# Supplementary material for: Dynamics of Antimicrobial Resistance and Genomic Epidemiology of Multidrug-Resistant Salmonella enterica Serovar Indiana ST17 from 2006 to 2017 in China
Source: mSystems. 2022 Jul 21;7(4):e00253-22. doi: 10.1128/msystems.00253-22 (PMC9426611; doi:10.1128/msystems.00253-22)
Supplement: TABLE S1 [file msystems.00253-22-s0001.pdf]

Table S1. Source and basic information of Salmonella indiana isolates

Strain nar	Collection laboratory	Country	Collection year	Source	Sex	age	Mutation site on gyrA	Mutation site on parC	Inc_types	AMP	CTX	IMI	MEM	GEN	STR	SUL	CHL	AZI	TET	NAL	CIP	CT
IndS1	National health commission Key Laboratory of Food Safety Risk Assessment	China	2006	chicken			S83F/D87G	T57S/S80R	IncHI2,IncHI2A,IncN	S	S	S	S	S	S	R	S	S	R	R	R	S
IndS2	National health commission Key Laboratory of Food Safety Risk Assessment	China	2006	chicken			S83F/D87G	T57S/S80R	IncHI2,IncHI2A,IncI1	R	R	S	S	S	S	R	S	S	S	R	R	S
IndS3	State Key Laboratory of Infectious Disease Prevention and Control	China	2007	feces	female	56	S83F/D87G	T57S/S80R	Col156	S	S	S	S	S	S	S	S	S	S	R	R	S
IndS4	National health commission Key Laboratory of Food Safety Risk Assessment	China	2007	chicken			S83F	T57S	IncFIB,IncN	S	S	S	S	S	S	R	S	R	S	R	S	S
IndS5	National health commission Key Laboratory of Food Safety Risk Assessment	China	2007	pork			S83F/D87G	T57S/S80R	IncHI2,IncHI2A,IncQ1	R	S	S	S	S	R	R	R	S	R	R	R	S
IndS6	State Key Laboratory of Infectious Disease Prevention and Control	China	2008	feces	female	45	S83F/D87G	T57S/S80R		S	S	S	S	S	S	S	S	S	S	R	R	S
IndS7	State Key Laboratory of Infectious Disease Prevention and Control	China	2008	feces	male	48	S83F/D87G	T57S/S80R	p0111	S	S	S	S	S	S	S	S	S	S	R	R	S
IndS8	National health commission Key Laboratory of Food Safety Risk Assessment	China	2008	duck			S83F/D87G	T57S/S80R	IncA/C2,IncI1,IncN	R	R	S	S	S	R	R	S	S	R	R	R	S
IndS9	National health commission Key Laboratory of Food Safety Risk Assessment	China	2008	chicken			S83F	T57S	IncA/C2	R	R	S	S	S	R	R	R	S	R	R	I	S
IndS10	National health commission Key Laboratory of Food Safety Risk Assessment	China	2008	pork			S83F/D87G	T57S/S80R	IncHI2,IncHI2A,IncQ1	R	S	S	S	S	R	R	R	S	R	R	R	S
IndS11	National health commission Key Laboratory of Food Safety Risk Assessment	China	2008	chicken			S83F/D87G	T57S/S80R	IncA/C2,IncHI2,IncHI2A,IncI1	R	R	S	S	I	R	R	R	S	R	R	R	S
IndS12	National health commission Key Laboratory of Food Safety Risk Assessment	China	2008	chicken			S83F/D87G	T57S/S80R		S	S	S	S	S	S	S	S	S	S	R	R	S
IndS13	National health commission Key Laboratory of Food Safety Risk Assessment	China	2008	chicken			S83F/D87G	T57S/S80R	IncHI2,IncHI2A,p0111	R	S	S	S	I	S	R	R	S	R	R	R	S
IndS14	National health commission Key Laboratory of Food Safety Risk Assessment	China	2008	chicken			S83F/D87G	T57S/S80R	p0111	S	S	S	S	S	S	S	S	S	S	R	R	S
IndS15	National health commission Key Laboratory of Food Safety Risk Assessment	China	2008	chicken			S83F/D87G	T57S/S80R	IncHI2,IncHI2A,IncN,IncQ1	R	R	S	S	S	R	R	R	S	R	R	R	S
IndS16	National health commission Key Laboratory of Food Safety Risk Assessment	China	2009	chicken			S83F/D87N	T57S/S80R	IncHI2,IncHI2A,IncX1	S	S	S	S	R	S	S	S	R	S	R	R	S
IndS17	National health commission Key Laboratory of Food Safety Risk Assessment	China	2009	chicken			S83F/D87N	T57S/S80R	IncHI2,IncHI2A,IncN,IncX1	R	R	S	S	S	S	R	R	R	R	R	R	S
IndS18	National health commission Key Laboratory of Food Safety Risk Assessment	China	2009	chicken			S83F/D87G	T57S/S80R	IncHI2,IncHI2A	R	S	S	S	I	S	R	R	S	S	R	R	S
IndS19	National health commission Key Laboratory of Food Safety Risk Assessment	China	2009	chicken			S83F/D87N	T57S/S80R		S	S	S	S	S	S	S	S	S	S	R	R	S
IndS20	National health commission Key Laboratory of Food Safety Risk Assessment	China	2009	chicken			S83F/D87G	T57S/S80R		S	S	S	S	S	S	S	S	S	S	R	R	S
IndS21	National health commission Key Laboratory of Food Safety Risk Assessment	China	2010	chicken			S83F/D87G	T57S/S80R	IncHI2,IncHI2A	S	S	S	S	S	S	S	R	S	R	R	R	S
IndS22	National health commission Key Laboratory of Food Safety Risk Assessment	China	2010	chicken			S83F/D87N	T57S/S80R	IncHI2,IncHI2A,IncX1,p0111	S	S	S	S	S	S	S	S	S	R	R	R	S
IndS23	National health commission Key Laboratory of Food Safety Risk Assessment	China	2010	chicken			S83F/D87G	T57S/S80R	IncI1	S	R	S	S	S	S	S	S	S	S	R	R	S
IndS24	National health commission Key Laboratory of Food Safety Risk Assessment	China	2010	egg			S83F/D87G	T57S/S80R	IncI1,p0111	S	R	S	S	S	S	S	S	S	S	R	R	S
IndS25	National health commission Key Laboratory of Food Safety Risk Assessment	China	2010	chicken			S83F/D87N	T57S/S80R	IncA/C2,IncHI2,IncHI2A,IncN	R	R	S	S	S	R	R	R	R	R	R	R	S
IndS26	National health commission Key Laboratory of Food Safety Risk Assessment	China	2010	chicken			S83F/D87N	T57S/S80R	IncA/C2	R	R	S	S	S	R	R	R	R	R	R	R	S
IndS27	National health commission Key Laboratory of Food Safety Risk Assessment	China	2010	Chicken manure			S83F/D87N	T57S/S80R	IncHI2,IncHI2A,IncX1	R	S	S	S	S	S	R	R	R	R	R	R	S
IndS28	National health commission Key Laboratory of Food Safety Risk Assessment	China	2010	chicken			S83F/D87N	T57S/S80R	IncHI2,IncHI2A,IncN,IncX1	R	R	S	S	R	R	S	R	R	R	R	R	S
IndS29	National health commission Key Laboratory of Food Safety Risk Assessment	China	2010	chicken			S83F/D87G	T57S/S80R	IncHI2,IncHI2A,IncN,IncQ1	R	R	S	S	I	R	R	R	S	S	R	R	S
IndS30	National health commission Key Laboratory of Food Safety Risk Assessment	China	2010	freshwater shell			S83F/D87N	T57S/S80R	IncHI2,IncHI2A,IncQ1	R	R	S	S	I	R	R	R	S	R	R	R	S
IndS31	National health commission Key Laboratory of Food Safety Risk Assessment	China	2010	chicken			S83F/D87N	T57S/S80R	IncHI2,IncHI2A,IncX1	R	S	S	S	R	S	R	R	R	R	R	R	S
IndS32	National health commission Key Laboratory of Food Safety Risk Assessment	China	2010	chicken			S83F/D87N	T57S/S80R	IncHI2,IncHI2A,IncN,IncQ1,IncX1	R	R	S	S	S	R	S	R	R	R	R	R	S
IndS33	National health commission Key Laboratory of Food Safety Risk Assessment	China	2010	chicken			S83F/D87G	T57S/S80R	IncI1	R	R	S	S	S	S	S	S	S	S	R	R	S
IndS34	State Key Laboratory of Infectious Disease Prevention and Control	China	2011	feces	female	37	S83F/D87N	T57S/S80R		S	S	S	S	S	S	S	S	S	S	R	R	S
IndS35	State Key Laboratory of Infectious Disease Prevention and Control	China	2011	feces	male	10	S83F/D87G	T57S/S80R	IncHI2,IncHI2A	R	R	S	S	I	S	R	R	R	S	R	R	S
IndS36	State Key Laboratory of Infectious Disease Prevention and Control	China	2011	feces	male	4	S83F/D87G	T57S/S80R	IncA/C2,IncHI2,IncHI2A,IncN	R	R	S	S	I	R	R	R	S	I	R	R	S
IndS37	State Key Laboratory of Infectious Disease Prevention and Control	China	2011	feces	male	3	S83F/D87G	T57S/S80R	IncA/C2	R	R	S	S	S	R	R	R	S	R	R	R	S
IndS38	State Key Laboratory of Infectious Disease Prevention and Control	China	2011	feces	male	10d	S83F/D87G	T57S/S80R	IncA/C2,IncHI2,IncHI2A	R	R	S	S	R	R	R	R	S	R	R	R	S
IndS39	State Key Laboratory of Infectious Disease Prevention and Control	China	2011	feces	female	1	S83F/D87G	T57S/S80R		S	S	S	S	S	S	R	R	S	S	R	R	S
IndS40	National health commission Key Laboratory of Food Safety Risk Assessment	China	2011	chicken			S83F/D87N	T57S/S80R	IncHI2,IncHI2A,IncX1	S	S	S	S	S	S	S	S	S	R	R	R	S
IndS43	National health commission Key Laboratory of Food Safety Risk Assessment	China	2011	marine fish			S83F/D87N	T57S/S80R		S	S	S	S	S	S	S	S	S	S	R	R	S
IndS44	National health commission Key Laboratory of Food Safety Risk Assessment	China	2011	chicken			S83F/D87N	T57S/S80R	Col156,IncQ1,IncX1	S	S	S	S	S	S	S	S	R	S	R	R	S
IndS45	National health commission Key Laboratory of Food Safety Risk Assessment	China	2011	duck			S83F/D87G	T57S/S80R		R	R	S	S	S	S	S	S	S	S	R	R	S
IndS46	National health commission Key Laboratory of Food Safety Risk Assessment	China	2011	chicken			S83F/D87N	T57S/S80R	IncHI2,IncHI2A,IncN,IncX1	R	R	S	S	S	S	R	R	R	S	R	R	S
IndS47	National health commission Key Laboratory of Food Safety Risk Assessment	China	2011	duck			S83F/D87N	T57S/S80R	IncHI2,IncHI2A,IncN	R	S	S	S	I	S	R	R	S	R	R	R	S
IndS48	National health commission Key Laboratory of Food Safety Risk Assessment	China	2011	duck			S83F/D87G	T57S/S80R	IncHI2,IncHI2A,IncQ1,IncY	R	R	S	S	I	R	R	R	S	S	R	R	S
IndS49	National health commission Key Laboratory of Food Safety Risk Assessment	China	2011	frog			S83F/D87G	T57S/S80R	IncHI2,IncHI2A	R	S	S	S	S	S	R	R	S	R	R	R	S
IndS50	National health commission Key Laboratory of Food Safety Risk Assessment	China	2011	chicken			S83F/D87N	T57S/S80R	Col156,IncHI2,IncHI2A,IncN,IncX1	R	R	S	S	S	S	R	R	R	R	R	R	S
IndS51	National health commission Key Laboratory of Food Safety Risk Assessment	China	2011	chicken			S83F/D87N	T57S/S80R	IncHI2,IncHI2A,IncX1	R	S	S	S	S	S	R	R	R	S	R	R	S
IndS52	National health commission Key Laboratory of Food Safety Risk Assessment	China	2011	chicken			S83F/D87N	T57S/S80R	IncX1	S	S	S	S	S	S	S	S	R	S	R	R	S
IndS53	National health commission Key Laboratory of Food Safety Risk Assessment	China	2011	chicken			S83F/D87G	T57S/S80R	IncFIB,IncHI2,IncHI2A,IncQ1	R	R	S	S	S	R	R	R	S	R	R	R	S
IndS54	National health commission Key Laboratory of Food Safety Risk Assessment	China	2011	chicken			S83F/D87N	T57S/S80R	IncHI2,IncHI2A,IncX1	S	S	S	S	S	S	S	S	R	S	R	R	S
IndS55	National health commission Key Laboratory of Food Safety Risk Assessment	China	2011	chicken			S83F/D87N	T57S/S80R	IncX1	S	S	S	S	S	S	S	S	R	S	R	R	S
IndS56	National health commission Key Laboratory of Food Safety Risk Assessment	China	2011	chicken			S83F/D87G	T57S/S80R	IncHI2,IncQ1	R	R	S	S	S	R	R	R	S	R	R	R	S
IndS57	National health commission Key Laboratory of Food Safety Risk Assessment	China	2011	chicken			S83F/D87N	T57S/S80R		R	R	S	S	S	S	S	S	S	S	R	R	S
IndS58	National health commission Key Laboratory of Food Safety Risk Assessment	China	2011	chicken			S83F/D87G	T57S/S80R	IncA/C2	R	S	S	S	R	R	R	R	S	R	R	R	S
IndS59	State Key Laboratory of Infectious Disease Prevention and Control	China	2012	blood	male	21d	S83F/D87N	T57S/S80R		R	R	S	S	S	S	S	S	S	S	R	R	S
IndS60	State Key Laboratory of Infectious Disease Prevention and Control	China	2012	CSF	female	11m	S83F/D87N	T57S/S80R		R	R	S	S	S	S	S	S	S	S	R	R	S
IndS61	State Key Laboratory of Infectious Disease Prevention and Control	China	2012	feces	male	21d	S83F/D87N	T57S/S80R		R	R	S	S	S	S	S	S	S	S	R	R	S
IndS62	State Key Laboratory of Infectious Disease Prevention and Control	China	2012	feces	male	21d	S83F/D87N	T57S/S80R	Col3M	R	R	S	S	S	S	S	S	S	S	R	R	S
IndS63	State Key Laboratory of Infectious Disease Prevention and Control	China	2014	feces	female	24	S83F/D87G	T57S/S80R	IncHI2,IncHI2A,IncQ1	R	S	S	S	S	R	R	R	S	R	R	R	S
IndS64	State Key Laboratory of Infectious Disease Prevention and Control	China	2012	feces	female	1	S83F/D87N	T57S/S80R	IncHI2,IncHI2A,IncN,IncQ1,IncX1	R	R	S	S	R	R	R	R	R	R	R	R	S
IndS65	National health commission Key Laboratory of Food Safety Risk Assessment	China	2012	chicken			S83F/D87N	T57S/S80R	IncHI2,IncHI2A,IncN	R	R	S	S	S	R	R	S	S	R	R	R	S

IndS66	National health commission Key Laboratory of Food Safety Risk Assessment	China	2012	chicken			S83F/D87N	T57S/S80R	IncHI2,IncHI2A,IncN,IncX1	R	S	S	S	S	S	R	R	R	R	R	R	S
IndS67	National health commission Key Laboratory of Food Safety Risk Assessment	China	2012	chicken			S83F/D87N	T57S/S80R	IncHI2,IncHI2A,IncN	R	R	S	S	R	S	R	R	R	R	R	R	S
IndS68	National health commission Key Laboratory of Food Safety Risk Assessment	China	2012	chicken			S83F/D87N	T57S/S80R	IncHI2,IncHI2A,IncX1	R	S	S	S	S	S	R	R	R	R	R	R	S
IndS69	National health commission Key Laboratory of Food Safety Risk Assessment	China	2012	chicken			S83F/D87N	T57S/S80R	Col156,IncHI2,IncHI2A,IncX1,p0111	R	R	S	S	R	S	R	R	R	R	R	R	S
IndS70	National health commission Key Laboratory of Food Safety Risk Assessment	China	2012	duck manure			D87G	T57S	IncHI2,IncHI2A,p0111	R	R	S	S	S	R	R	R	S	S	R	R	S
IndS71	National health commission Key Laboratory of Food Safety Risk Assessment	China	2012	duck manure			D87G	T57S	IncHI2,IncHI2A,p0111	R	R	S	S	S	R	R	R	S	S	R	R	S
IndS72	National health commission Key Laboratory of Food Safety Risk Assessment	China	2012	chicken manure			S83F/D87N	T57S/S80R	IncHI2A,IncN	S	S	S	S	S	R	R	S	R	R	R	R	S
IndS73	National health commission Key Laboratory of Food Safety Risk Assessment	China	2012	chicken			S83F/D87N	T57S/S80R		S	S	S	S	S	S	S	S	S	S	R	R	S
IndS74	National health commission Key Laboratory of Food Safety Risk Assessment	China	2012	chicken			S83F/D87G	T57S/S80R	IncA/C2	S	S	S	S	S	R	R	S	S	R	R	R	S
IndS76	National health commission Key Laboratory of Food Safety Risk Assessment	China	2012	chicken			S83F/D87N	T57S/S80R	IncA/C2	R	R	S	S	S	R	R	R	R	R	R	R	S
IndS77	National health commission Key Laboratory of Food Safety Risk Assessment	China	2012	chicken			S83F/D87N	T57S/S80R	IncQ1,IncX1	R	R	S	S	I	R	R	R	R	R	R	R	S
IndS79	National health commission Key Laboratory of Food Safety Risk Assessment	China	2012	chicken			S83F/D87N	T57S/S80R		S	S	S	S	S	S	S	S	S	S	R	R	S
IndS80	National health commission Key Laboratory of Food Safety Risk Assessment	China	2012	duck			S83F/D87G	T57S/S80R	IncHI2,IncHI2A,p0111	R	S	S	S	I	S	R	R	S	S	R	R	S
IndS81	National health commission Key Laboratory of Food Safety Risk Assessment	China	2012	chicken			S83F/D87N	T57S/S80R	IncHI2,IncHI2A,IncX1,p0111	R	R	S	S	R	S	R	R	R	R	R	R	S
IndS82	National health commission Key Laboratory of Food Safety Risk Assessment	China	2012	chicken			S83F/D87N	T57S/S80R	Col156,IncHI2,IncHI2A,IncN,IncQ1,IncX1	R	R	S	S	I	R	R	R	R	R	R	R	S
IndS83	State Key Laboratory of Infectious Disease Prevention and Control	China	2013	feces	male	26	S83F/D87N	T57S/S80R	IncHI2,IncHI2A,IncN	R	S	S	S	S	R	R	R	R	I	R	R	S
IndS84	State Key Laboratory of Infectious Disease Prevention and Control	China	2013	feces	female	1	S83F/D87N	T57S/S80R	IncHI2,IncHI2A,IncN,IncX1	R	R	S	S	I	R	R	R	S	R	R	R	S
IndS85	State Key Laboratory of Infectious Disease Prevention and Control	China	2013	feces	female	31	S83F/D87N	T57S/S80R	IncFIA,IncFIB,IncFII	R	R	S	S	R	R	R	R	R	S	R	R	S
IndS86	State Key Laboratory of Infectious Disease Prevention and Control	China	2013	feces	male	8m	S83F/D87N	T57S/S80R	IncX1	R	S	S	S	R	S	S	R	R	S	R	R	S
IndS87	State Key Laboratory of Infectious Disease Prevention and Control	China	2013	feces	male	1	S83F/D87N	T57S/S80R		S	S	S	S	S	S	S	S	S	S	R	R	S
IndS88	National health commission Key Laboratory of Food Safety Risk Assessment	China	2013	chicken			S83F/D87N	T57S/S80R		S	S	S	S	S	S	S	S	S	S	R	R	S
IndS89	National health commission Key Laboratory of Food Safety Risk Assessment	China	2013	duck			S83F/D87N	T57S/S80R	IncHI2,IncHI2A	R	R	S	S	I	S	R	R	S	R	R	R	S
IndS90	National health commission Key Laboratory of Food Safety Risk Assessment	China	2013	duck			S83F/D87N	T57S/S80R	IncHI2,IncHI2A,IncN,IncX1	R	S	S	S	R	S	R	R	R	S	R	R	S
IndS91	National health commission Key Laboratory of Food Safety Risk Assessment	China	2013	chicken			S83F/D87N	T57S/S80R	IncHI2,IncHI2A	R	R	S	S	R	S	R	R	S	R	R	R	S
IndS92	National health commission Key Laboratory of Food Safety Risk Assessment	China	2013	chicken			S83F/D87N	T57S/S80R	IncA/C2	R	R	S	S	S	R	R	R	S	R	R	R	S
IndS93	National health commission Key Laboratory of Food Safety Risk Assessment	China	2013	seawater shell			S83F/D87G	T57S/S80R		R	S	S	S	R	S	S	S	S	S	R	R	S
IndS94	National health commission Key Laboratory of Food Safety Risk Assessment	China	2013	seawater shell			S83F/D87G	T57S/S80R	IncHI2,IncHI2A,IncQ1	R	S	S	S	R	R	R	R	S	S	R	R	S
IndS95	National health commission Key Laboratory of Food Safety Risk Assessment	China	2013	frog			S83F/D87N	T57S/S80R	IncHI2,IncHI2A,IncN,IncX1	S	S	S	S	S	S	S	S	R	R	R	R	S
IndS96	National health commission Key Laboratory of Food Safety Risk Assessment	China	2012	pork			S83F/D87N	T57S/S80R	IncHI2,IncHI2A,IncN,IncX1	S	S	S	S	S	S	S	S	R	R	R	R	S
IndS97	National health commission Key Laboratory of Food Safety Risk Assessment	China	2012	chicken			S83F/D87N	T57S/S80R	IncHI2,IncHI2A,IncQ1,IncX1	R	R	S	S	R	R	R	R	R	R	R	R	S
IndS98	National health commission Key Laboratory of Food Safety Risk Assessment	China	2013	chicken			S83F/D87N	T57S/S80R	IncHI2,IncHI2A,IncN,IncQ1,IncX1	R	R	S	S	R	R	S	R	R	R	R	R	S
IndS99	National health commission Key Laboratory of Food Safety Risk Assessment	China	2013	pork			S83F/D87N	T57S/S80R	IncHI2,IncHI2A,IncN,IncX1	R	S	S	S	R	S	R	R	R	R	R	R	S
IndS100	National health commission Key Laboratory of Food Safety Risk Assessment	China	2013	chicken			S83F/D87N	T57S/S80R	Col3M,IncHI2,IncHI2A,IncN,IncX1	S	S	S	S	S	S	S	S	R	S	R	R	S
IndS101	National health commission Key Laboratory of Food Safety Risk Assessment	China	2013	pork			S83F/D87N	T57S/S80R	IncHI2,IncHI2A,IncN,IncX1	R	S	S	S	R	S	R	R	R	R	R	R	S
IndS102	National health commission Key Laboratory of Food Safety Risk Assessment	China	2013	duck			S83F/D87N	T57S/S80R	IncHI2,IncHI2A,IncN	R	R	S	S	S	S	R	R	S	S	R	R	S
IndS103	National health commission Key Laboratory of Food Safety Risk Assessment	China	2013	chicken			S83F/D87N	T57S/S80R	IncHI2,IncHI2A,IncX1	R	R	S	S	R	S	R	R	R	R	R	R	S
IndS104	National health commission Key Laboratory of Food Safety Risk Assessment	China	2013	chicken			S83F/D87N	T57S/S80R	IncHI2,IncX1	R	R	S	S	R	S	R	R	R	S	R	R	S
IndS105	National health commission Key Laboratory of Food Safety Risk Assessment	China	2013	chicken			S83F/D87N	T57S/S80R	IncHI2,IncHI2A,IncX1	R	S	S	S	S	S	R	R	R	R	R	R	S
IndS106	National health commission Key Laboratory of Food Safety Risk Assessment	China	2013	chicken			S83F/D87G	T57S/S80R	Col156,IncHI2,IncHI2A,IncN	R	S	S	S	I	S	R	R	S	R	R	R	S
IndS107	National health commission Key Laboratory of Food Safety Risk Assessment	China	2013	chicken			S83F/D87N	T57S/S80R	IncHI2,IncHI2A,IncX1	R	S	S	S	S	S	S	S	R	R	R	R	S
IndS108	National health commission Key Laboratory of Food Safety Risk Assessment	China	2013	chicken			S83F/D87N	T57S/S80R	IncA/C2	R	R	S	S	S	R	R	R	R	R	R	R	S
IndS109	State Key Laboratory of Infectious Disease Prevention and Control	China	2014	feces	female	40	S83F/D87G	T57S/S80R		S	S	S	S	S	S	S	S	S	S	R	R	S
IndS111	State Key Laboratory of Infectious Disease Prevention and Control	China	2009	feces			S83F/D87G	T57S/S80R		S	S	S	S	S	S	S	S	S	S	R	R	S
IndS112	National health commission Key Laboratory of Food Safety Risk Assessment	China	2015	chicken			S83F/D87N	T57S/S80R		R	R	S	S	R	R	R	R	S	R	R	R	S
IndS113	National health commission Key Laboratory of Food Safety Risk Assessment	China	2014	chicken			S83F/D87N	T57S/S80R	IncHI2,IncHI2A,IncQ1,IncX1	R	R	S	S	I	R	R	R	S	R	R	R	S
IndS114	National health commission Key Laboratory of Food Safety Risk Assessment	China	2014	pork			S83F/D87G	T57S/S80R	IncI1,IncX1	R	R	S	S	S	S	R	R	S	R	R	R	S
IndS115	National health commission Key Laboratory of Food Safety Risk Assessment	China	2014	chicken			S83F/D87N	T57S/S80R	IncN	R	S	S	S	S	S	R	R	S	S	R	R	S
IndS116	National health commission Key Laboratory of Food Safety Risk Assessment	China	2014	chicken			S83F/D87N	T57S/S80R	IncHI2,IncHI2A,IncN,IncX1	R	R	S	S	R	S	R	R	R	R	R	R	S
IndS117	National health commission Key Laboratory of Food Safety Risk Assessment	China	2014	chicken manure			S83F/D87G	T57S/S80R	IncA/C2,IncQ1	R	S	S	S	R	R	R	R	S	R	R	R	S
IndS118	National health commission Key Laboratory of Food Safety Risk Assessment	China	2014	duck manure			D87G	T57S	p0111	R	R	S	S	S	R	R	R	S	R	R	S	S
IndS119	National health commission Key Laboratory of Food Safety Risk Assessment	China	2014	frozen food			S83F/D87N	T57S/S80R	IncHI2,IncHI2A,IncN,IncX1	R	R	S	S	S	R	R	R	R	R	R	R	S
IndS121	National health commission Key Laboratory of Food Safety Risk Assessment	China	2014	dairy products			S83F/D87N	T57S/S80R	IncX1	S	S	S	S	S	S	S	S	S	S	R	R	S
IndS122	National health commission Key Laboratory of Food Safety Risk Assessment	China	2014	chicken			S83F/D87N	T57S/S80R	IncN	R	R	S	S	S	S	R	R	S	R	R	R	S
IndS123	National health commission Key Laboratory of Food Safety Risk Assessment	China	2014	chicken			S83F/D87G	T57S/S80R		S	S	S	S	S	S	S	S	S	S	R	R	S
IndS124	National health commission Key Laboratory of Food Safety Risk Assessment	China	2014	chicken			S83F/D87N	T57S/S80R		S	S	S	S	S	S	R	R	S	S	R	R	S
IndS125	National health commission Key Laboratory of Food Safety Risk Assessment	China	2014	chicken			S83F/D87N	T57S/S80R	Col156,IncHI2,IncHI2A,IncX1	R	S	S	S	S	S	S	R	S	R	R	R	S
IndS126	National health commission Key Laboratory of Food Safety Risk Assessment	China	2014	chicken			S83F/D87N	T57S/S80R	ColpVC,IncA/C2,IncHI2,IncHI2A	R	R	S	S	S	R	R	R	R	R	R	R	S
IndS127	State Key Laboratory of Infectious Disease Prevention and Control	China	2015	feces	female	55	S83F/D87G	T57S/S80R		R	S	S	S	R	S	R	R	S	R	R	R	S
IndS128	State Key Laboratory of Infectious Disease Prevention and Control	China	2015	feces	female	13	S83F/D87G	T57S/S80R	IncHI2,IncHI2A	S	R	S	S	S	S	R	R	R	S	R	R	S
IndS129	State Key Laboratory of Infectious Disease Prevention and Control	China	2015	feces	male	6	S83F/D87N	T57S/S80R	Col156,IncHI2,IncHI2A,IncN	R	R	S	S	I	R	R	R	R	S	R	R	S
IndS130	State Key Laboratory of Infectious Disease Prevention and Control	China	2015	feces	male	6	S83F/D87N	T57S/S80R	IncHI2,IncHI2A,IncN	R	R	S	S	S	R	R	S	R	S	R	R	S
IndS131	State Key Laboratory of Infectious Disease Prevention and Control	China	2015	feces	male	6	S83F/D87N	T57S/S80R	IncHI2,IncHI2A,IncN	R	S	S	S	I	R	R	R	R	S	R	R	S
IndS132	State Key Laboratory of Infectious Disease Prevention and Control	China	2015	feces	female	57	S83F/D87N	T57S/S80R	IncHI2,IncHI2A,IncN,IncQ1,IncX1	R	R	S	S	I	R	R	R	R	S	R	R	S
IndS133	State Key Laboratory of Infectious Disease Prevention and Control	China	2015	feces	male	4m	D87G	T57S		S	S	S	S	S	R	R	R	S	R	R	S	S
IndS134	State Key Laboratory of Infectious Disease Prevention and Control	China	2014	feces	male	68	S83F/D87N	T57S/S80R		R	R	S	S	I	R	S	R	S	R	R	R	S
IndS135	State Key Laboratory of Infectious Disease Prevention and Control	China	2015	feces	male	1	S83F/D87G	T57S/S80R		R	S	S	S	S	S	R	R	S	R	R	R	S

IndS136	National health commission Key Laboratory of Food Safety Risk Assessment	China	2015	chicken			S83F/D87N	T57S/S80R		S	S	S	S	S	S	S	S	S	S	R	R	S
IndS137	National health commission Key Laboratory of Food Safety Risk Assessment	China	2015	chicken			S83F/D87N	T57S/S80R	IncHI2,IncHI2A,IncQ1,IncX1	R	R	S	S	I	R	R	R	R	S	R	R	S
IndS138	National health commission Key Laboratory of Food Safety Risk Assessment	China	2015	chicken			S83F/D87N	T57S/S80R		R	S	S	S	R	S	R	R	R	S	R	R	S
IndS139	National health commission Key Laboratory of Food Safety Risk Assessment	China	2015	chicken			S83F/D87N	T57S/S80R	IncHI2,IncHI2A,IncN,p0111	R	R	S	S	I	S	R	R	S	S	R	R	S
IndS140	National health commission Key Laboratory of Food Safety Risk Assessment	China	2015	chicken			S83F/D87N	T57S/S80R	IncHI2,IncHI2A,IncN,p0111	R	R	S	S	S	S	R	R	S	R	R	R	S
IndS141	National health commission Key Laboratory of Food Safety Risk Assessment	China	2015	chicken			S83F/D87N	T57S/S80R	IncHI2,IncHI2A,IncN,p0111	R	R	S	S	S	S	R	R	S	S	R	R	S
IndS142	National health commission Key Laboratory of Food Safety Risk Assessment	China	2015	chicken			S83F/D87N	T57S/S80R	IncX1	R	R	S	S	I	S	R	R	R	S	R	R	S
IndS143	National health commission Key Laboratory of Food Safety Risk Assessment	China	2015	chicken			S83F/D87G	T57S/S80R		R	S	S	S	R	S	R	R	S	R	R	R	S
IndS144	National health commission Key Laboratory of Food Safety Risk Assessment	China	2015	chicken			S83F/D87N	T57S/S80R	IncX1	R	S	S	S	S	S	R	R	R	R	R	R	S
IndS145	National health commission Key Laboratory of Food Safety Risk Assessment	China	2015	chicken			S83F/D87N	T57S/S80R	IncX1	R	S	S	S	S	S	R	R	R	R	R	R	S
IndS146	National health commission Key Laboratory of Food Safety Risk Assessment	China	2015	Chicken			S83F/D87G	T57S/S80R		R	S	S	S	S	S	R	R	S	R	R	R	S
IndS147	National health commission Key Laboratory of Food Safety Risk Assessment	China	2015	Chicken			S83F/D87G	T57S/S80R		R	S	S	S	R	S	R	R	S	R	R	R	S
IndS148	National health commission Key Laboratory of Food Safety Risk Assessment	China	2015	Chicken			S83F/D87G	T57S/S80R		R	S	S	S	R	S	R	R	S	R	R	R	S
IndS149	National health commission Key Laboratory of Food Safety Risk Assessment	China	2015	Chicken			D87G	T57S	p0111	S	S	S	S	S	R	R	R	S	R	R	S	S
IndS150	National health commission Key Laboratory of Food Safety Risk Assessment	China	2015	Chicken			S83F/D87G	T57S/S80R		R	S	S	S	R	S	R	R	S	R	R	R	S
IndS151	National health commission Key Laboratory of Food Safety Risk Assessment	China	2015	Chicken			S83F/D87G	T57S/S80R	IncFIB,IncHI2,IncHI2A	R	R	S	S	R	R	R	R	S	R	R	R	S
IndS152	National health commission Key Laboratory of Food Safety Risk Assessment	China	2015	Chicken			D87G	T57S	IncHI2,IncHI2A,p0111	R	R	S	S	S	S	R	R	S	R	R	S	S
IndS153	National health commission Key Laboratory of Food Safety Risk Assessment	China	2015	duck			S83F/D87N	T57S/S80R		S	S	S	S	I	S	R	R	S	S	R	R	S
IndS154	National health commission Key Laboratory of Food Safety Risk Assessment	China	2015	chicken			S83F/D87N	T57S/S80R	IncHI2,IncHI2A,IncN,IncX1	R	R	S	S	R	S	R	R	R	R	R	R	S
IndS155	National health commission Key Laboratory of Food Safety Risk Assessment	China	2015	chicken			S83F/D87N	T57S/S80R	IncHI2,IncHI2A,IncX1	R	R	S	S	S	S	R	R	S	R	R	R	S
IndS158	National health commission Key Laboratory of Food Safety Risk Assessment	China	2015	freshwater shell			S83F/D87G	T57S/S80R		S	S	S	S	S	S	S	S	S	S	R	R	S
IndS159	National health commission Key Laboratory of Food Safety Risk Assessment	China	2015	chicken			S83F/D87N	T57S/S80R	IncHI2,IncHI2A	R	R	S	S	S	S	R	R	S	R	R	R	S
IndS160	National health commission Key Laboratory of Food Safety Risk Assessment	China	2015	chicken			S83F/D87G	T57S/S80R	IncHI2,IncHI2A	R	S	S	S	R	S	R	R	S	S	R	R	S
IndS161	National health commission Key Laboratory of Food Safety Risk Assessment	China	2015	bean products			S83F/D87N	T57S/S80R		S	S	S	S	S	S	S	S	S	S	R	R	S
IndS162	National health commission Key Laboratory of Food Safety Risk Assessment	China	2015	chicken			S83F/D87N	T57S/S80R	IncHI2,IncHI2A	R	S	S	S	I	S	R	R	S	S	R	R	S
IndS163	National health commission Key Laboratory of Food Safety Risk Assessment	China	2015	chicken			S83F/D87G	T57S/S80R	IncHI2,IncHI2A,IncQ1,p0111	R	R	S	S	S	R	R	R	S	S	R	R	S
IndS164	National health commission Key Laboratory of Food Safety Risk Assessment	China	2015	pork			S83F/D87N	T57S/S80R	IncFII,IncHI2,IncHI2A,IncN,IncR	R	R	S	S	R	S	R	R	R	R	R	R	S
IndS165	National health commission Key Laboratory of Food Safety Risk Assessment	China	2015	chicken			S83F/D87N	T57S/S80R	Col156,IncHI2,IncHI2A,IncN,IncX1,p0111	S	S	S	S	S	S	S	S	R	S	R	R	S
IndS166	National health commission Key Laboratory of Food Safety Risk Assessment	China	2015	chicken			S83F/D87N	T57S/S80R	IncFII,IncR	R	R	S	S	R	S	R	R	R	R	R	R	S
IndS167	State Key Laboratory of Infectious Disease Prevention and Control	China	2015	feces	female	60	S83F/D87G	T57S/S80R		R	S	S	S	R	S	R	R	S	R	R	R	S
IndS168	State Key Laboratory of Infectious Disease Prevention and Control	China	2016	feces	male	3	S83F/D87N	T57S/S80R		R	R	S	S	R	R	R	R	R	R	R	R	S
IndS169	State Key Laboratory of Infectious Disease Prevention and Control	China	2015	feces	female	30	S83F/D87N	T57S/S80R		R	R	S	S	S	S	S	S	S	S	R	R	S
IndS170	State Key Laboratory of Infectious Disease Prevention and Control	China	2015	feces	female	28	S83F/D87G	T57S/S80R		R	S	S	S	R	S	R	R	S	R	R	R	S
IndS171	State Key Laboratory of Infectious Disease Prevention and Control	China	2015	feces	female	31	S83F/D87G	T57S/S80R		R	S	S	S	R	S	R	R	S	R	R	R	S
IndS172	State Key Laboratory of Infectious Disease Prevention and Control	China	2016	feces	male	38	S83F/D87N	T57S/S80R	Col156,IncHI2,IncHI2A,IncN	R	S	S	S	R	S	R	R	S	R	R	R	S
IndS173	State Key Laboratory of Infectious Disease Prevention and Control	China	2016	feces	female	62	S83F/D87N	T57S/S80R		R	R	S	S	S	R	R	R	S	R	R	R	S
IndS174	State Key Laboratory of Infectious Disease Prevention and Control	China	2016	feces	male	49	S83F/D87N	T57S/S80R	IncHI2,IncHI2A,IncN	R	R	S	S	R	R	R	R	R	R	R	R	S
IndS175	State Key Laboratory of Infectious Disease Prevention and Control	China	2016	feces	male	4	S83F/D87G	T57S/S80R	IncFIB,p0111	R	R	S	S	I	S	R	R	S	R	R	R	S
IndS176	State Key Laboratory of Infectious Disease Prevention and Control	China	2016	feces	male	1	S83F/D87G	T57S/S80R	ColpVC,IncFIA,IncHI1A,IncHI1B	S	S	S	S	S	S	S	S	S	S	R	R	S
IndS177	State Key Laboratory of Infectious Disease Prevention and Control	China	2016	feces	male	1	S83F/D87G	T57S/S80R	ColpVC,IncFIA,IncHI1A,IncHI1B	R	S	S	S	R	S	R	R	S	R	R	R	S
IndS178	State Key Laboratory of Infectious Disease Prevention and Control	China	2016	feces	male	1	S83F/D87G	T57S/S80R	IncFIA,IncFII,IncHI1A,IncHI1B	R	R	S	S	R	S	R	R	S	R	R	R	S
IndS179	State Key Laboratory of Infectious Disease Prevention and Control	China	2016	feces	male	1	S83F/D87G	T57S/S80R		S	S	S	S	S	S	S	S	S	S	R	R	S
IndS180	State Key Laboratory of Infectious Disease Prevention and Control	China	2016	feces	male	1	S83F/D87G	T57S/S80R	IncHI2,IncHI2A,IncQ1	R	R	S	S	I	R	R	R	S	R	R	R	S
IndS181	State Key Laboratory of Infectious Disease Prevention and Control	China	2016	feces	male	1	S83F/D87G	T57S/S80R	IncHI2,IncHI2A,IncN,IncQ1	R	R	S	S	I	R	R	R	S	R	R	R	S
IndS182	State Key Laboratory of Infectious Disease Prevention and Control	China	2016	feces	female	10m	S83F/D87G	T57S/S80R	IncHI2,IncHI2A,IncN,IncQ1	R	R	S	S	I	R	R	R	S	R	R	R	S
IndS183	State Key Laboratory of Infectious Disease Prevention and Control	China	2016	feces	female	10m	S83F/D87G	T57S/S80R	IncHI2,IncHI2A,IncN,IncQ1	R	R	S	S	R	R	R	R	S	S	R	R	S
IndS184	State Key Laboratory of Infectious Disease Prevention and Control	China	2016	feces	female	1m	S83F/D87G	T57S/S80R	IncFIA,IncHI1A,IncHI1B	R	S	S	S	R	S	R	R	S	R	R	R	S
IndS185	State Key Laboratory of Infectious Disease Prevention and Control	China	2007	feces	female	36	D87G	T57S	IncHI2,IncHI2A,IncN,IncQ1	S	S	S	S	S	R	R	S	S	S	R	S	S
IndS186	State Key Laboratory of Infectious Disease Prevention and Control	China	2010	feces	male	34	S83F/D87N	T57S/S80R		I	R	S	S	S	S	S	S	S	S	R	R	S
IndS187	State Key Laboratory of Infectious Disease Prevention and Control	China	2010	feces	male	2	S83F/D87N	T57S/S80R		R	R	S	S	S	S	S	S	S	S	R	R	S
IndS188	State Key Laboratory of Infectious Disease Prevention and Control	China	2011	feces	female	1	S83F/D87N	T57S/S80R	IncA/C2	R	R	S	S	S	R	R	R	S	R	R	R	S
IndS189	State Key Laboratory of Infectious Disease Prevention and Control	China	2011	feces	male	2m	S83F/D87N	T57S/S80R	IncA/C2	R	R	S	S	S	R	R	R	R	R	R	R	S
IndS190	State Key Laboratory of Infectious Disease Prevention and Control	China	2011	feces	male	5m	S83F/D87N	T57S/S80R		R	R	S	S	S	R	R	R	S	R	R	R	S
IndS191	State Key Laboratory of Infectious Disease Prevention and Control	China	2013	feces	female	1	S83F/D87N	T57S/S80R	IncHI2,IncHI2A,IncX1,p0111	R	R	S	S	S	S	R	R	S	R	R	R	S
IndS192	State Key Laboratory of Infectious Disease Prevention and Control	China	2014	feces	female	1	S83F/D87N	T57S/S80R	IncHI2,IncHI2A,IncX1	R	R	S	S	R	S	R	R	R	S	R	R	S
IndS194	State Key Laboratory of Infectious Disease Prevention and Control	China	2014	feces	male	1	S83F/D87N	T57S/S80R	IncFIA,IncFIB,IncFII,IncN,IncQ1	R	R	S	S	R	R	S	S	R	R	R	R	S
IndS195	State Key Laboratory of Infectious Disease Prevention and Control	China	2016	feces	female	29	S83F/D87N	T57S/S80R		R	R	S	S	I	R	R	R	S	R	R	R	S
IndS196	State Key Laboratory of Infectious Disease Prevention and Control	China	2016	feces	male	36	S83F/D87G	T57S/S80R	ColpVC	S	S	S	S	S	S	S	S	S	S	R	R	S
IndS197	State Key Laboratory of Infectious Disease Prevention and Control	China	2016	feces	male	40	S83F/D87G	T57S/S80R		R	S	S	S	R	S	R	R	S	R	R	R	S
IndS198	State Key Laboratory of Infectious Disease Prevention and Control	China	2016	feces	female	42	S83F/D87G	T57S/S80R		R	S	S	S	R	S	R	R	S	R	R	R	S
IndS199	State Key Laboratory of Infectious Disease Prevention and Control	China	2016	feces	female	34	S83F/D87G	T57S/S80R		R	S	S	S	I	S	R	R	S	R	R	R	S
IndS200	State Key Laboratory of Infectious Disease Prevention and Control	China	2016	feces	female	41	S83F/D87G	T57S/S80R		R	S	S	S	R	S	R	R	S	S	R	R	S
IndS201	State Key Laboratory of Infectious Disease Prevention and Control	China	2016	feces	female	61	S83F/D87G	T57S/S80R	IncHI2,IncHI2A	R	S	S	S	S	S	R	R	S	S	R	R	S
IndS202	National health commission Key Laboratory of Food Safety Risk Assessment	China	2015/2016	chicken			S83F/D87N	T57S/S80R	IncHI2,IncHI2A,IncN,IncQ1	R	R	S	S	R	R	R	R	R	R	R	R	S
IndS203	National health commission Key Laboratory of Food Safety Risk Assessment	China	2015/2016	chicken			S83F/D87N	T57S/S80R	IncHI2,IncHI2A,IncN,IncX1	R	R	S	S	R	S	R	R	R	R	R	R	S
IndS204	National health commission Key Laboratory of Food Safety Risk Assessment	China	2015/2016	chicken			S83F/D87N	T57S/S80R		R	R	S	S	I	R	R	R	S	R	R	R	S

IndS205	National health commission Key Laboratory of Food Safety Risk Assessment	China	2015/2016	chicken			S83F/D87N	T57S/S80R	IncX1	R	R	S	S	R	S	R	R	R	R	R	R	S
IndS206	State Key Laboratory of Infectious Disease Prevention and Control	China	2013	feces	male	27	S83F/D87G	T57S/S80R		R	S	S	S	I	S	R	R	S	R	R	R	S
IndS207	State Key Laboratory of Infectious Disease Prevention and Control	China	2013	feces	male	33	S83F/D87N	T57S/S80R		R	S	S	S	S	S	R	R	S	R	R	R	S
IndS208	State Key Laboratory of Infectious Disease Prevention and Control	China	2014	feces	female	32	S83F/D87G	T57S/S80R		R	S	S	S	S	S	R	R	S	R	R	R	S
IndS209	State Key Laboratory of Infectious Disease Prevention and Control	China	2014	feces	female	24	S83F/D87G	T57S/S80R	IncHI2,IncHI2A	S	S	S	S	S	S	S	R	S	R	R	R	S
IndS210	State Key Laboratory of Infectious Disease Prevention and Control	China	2014	feces	male	50	S83F/D87G	T57S/S80R	IncHI2,IncHI2A	R	R	S	S	R	S	R	R	R	R	R	R	S
IndS211	State Key Laboratory of Infectious Disease Prevention and Control	China	2014	feces	female	24	S83F/D87G	T57S/S80R	IncHI2,IncHI2A,IncQ1	R	S	S	S	S	R	R	R	S	R	R	R	S
IndS212	State Key Laboratory of Infectious Disease Prevention and Control	China	2014	feces	female	32	S83F/D87G	T57S/S80R		R	S	S	S	S	S	R	R	S	R	R	R	S
IndS213	State Key Laboratory of Infectious Disease Prevention and Control	China	2014	feces	female	41	S83F/D87G	T57S/S80R		R	S	S	S	I	S	R	R	S	R	R	R	S
s10100	State Key Laboratory of Infectious Disease Prevention and Control	China	2010	feces	male	7	S83F/D87G	T57S/S80R	IncA/C2,IncN	R	R	S	S	R	I	R	R	S	R	R	R	S
s11011	State Key Laboratory of Infectious Disease Prevention and Control	China	2011	feces	female	22	S83F/D87G	T57S/S80R	IncHI2,IncHI2A	R	R	S	S	I	I	R	R	S	R	R	R	S
s11012	State Key Laboratory of Infectious Disease Prevention and Control	China	2011	feces	female	50	S83F/D87G	T57S/S80R	IncHI2,IncHI2A	R	R	S	S	I	I	R	R	S	R	R	R	S
s11033	State Key Laboratory of Infectious Disease Prevention and Control	China	2011	feces	male	1	S83F/D87N	T57S/S80R	IncHI2,IncHI2A,IncN,IncQ1,IncX1	R	R	S	S	R	R	R	R	R	R	R	R	S
s11066	State Key Laboratory of Infectious Disease Prevention and Control	China	2011	feces	male	0	S83F/D87G	T57S/S80R	Col3M,IncHI2,IncHI2A,p0111	R	R	S	S	S	I	R	R	S	R	R	R	S
s11091	State Key Laboratory of Infectious Disease Prevention and Control	China	2011	feces	female	28	S83F/D87N	T57S/S80R	IncHI2,IncHI2A,IncN,IncQ1,IncX1	R	R	S	S	R	I	S	R	R	R	R	R	S
s11092	State Key Laboratory of Infectious Disease Prevention and Control	China	2011	feces	male	0	S83F/D87N	T57S/S80R	IncHI2,IncHI2A,IncN,IncQ1,IncX1	R	R	S	S	R	I	S	R	R	R	R	R	S
s11093	State Key Laboratory of Infectious Disease Prevention and Control	China	2011	feces	male	36	S83F/D87N	T57S/S80R	IncHI2,IncHI2A,IncN,IncQ1,IncX1	R	R	S	S	R	I	S	R	R	R	R	R	S
s11094	State Key Laboratory of Infectious Disease Prevention and Control	China	2011	feces	male	1	S83F/D87N	T57S/S80R	IncHI2,IncHI2A,IncN,IncQ1,IncX1	R	R	S	S	R	I	S	R	R	R	R	R	S
s11229	State Key Laboratory of Infectious Disease Prevention and Control	China	2011	feces	male	0	S83F/D87G	T57S/S80R	IncHI2,IncHI2A	R	R	S	S	I	S	R	R	R	R	R	R	S
s12120	State Key Laboratory of Infectious Disease Prevention and Control	China	2012	feces	male	0.4	S83F/D87N	T57S/S80R	IncHI2,IncHI2A,IncN,IncQ1,IncX1	R	R	S	S	R	R	S	R	R	R	R	R	S
s12150	State Key Laboratory of Infectious Disease Prevention and Control	China	2012	feces	female	3.7	S83F/D87N	T57S/S80R	Col156,ColpVC,IncHI2,IncHI2A,IncX1,p0	R	S	S	S	S	S	R	R	R	R	R	R	S
s12151	State Key Laboratory of Infectious Disease Prevention and Control	China	2007	feces	male	19	S83F/D87N	T57S/S80R		R	R	S	S	R	S	R	R	R	S	R	R	S
s12159	State Key Laboratory of Infectious Disease Prevention and Control	China	2007	feces	female	57	S83F/D87G	T57S/S80R	IncA/C2,IncN,IncQ1	R	R	S	S	R	R	S	R	R	R	R	R	S
s12171	State Key Laboratory of Infectious Disease Prevention and Control	China	2007	feces	male	16	S83F/D87G	T57S/S80R	IncHI2,IncHI2A	R	R	S	S	R	S	R	R	R	S	R	R	S
s12177	State Key Laboratory of Infectious Disease Prevention and Control	China	2007	feces	female	8m	S83F/D87G	T57S/S80R	IncHI2,IncHI2A,IncN,IncQ1	R	R	S	S	R	R	R	R	S	R	R	R	S
s12188	State Key Laboratory of Infectious Disease Prevention and Control	China	2009	feces	female	1	S83F/D87N	T57S/S80R	Col156,IncHI2,IncHI2A,IncN,IncX1	S	S	S	S	R	S	S	S	R	R	R	R	S
s12193	State Key Laboratory of Infectious Disease Prevention and Control	China	2012	feces	female	1.3	S83F/D87G	T57S/S80R	Col156,Col8282,IncHI2,IncHI2A,IncI1	R	R	S	S	R	S	R	R	S	R	R	R	S
s12194	State Key Laboratory of Infectious Disease Prevention and Control	China	2012	feces	female	1.3	S83F/D87G	T57S/S80R	Col156,Col8282,IncHI2,IncHI2A,IncI1	R	R	S	S	R	S	R	R	S	R	R	R	S
s12197	State Key Laboratory of Infectious Disease Prevention and Control	China	2012	feces	male	1.5	S83F/D87G	T57S/S80R	IncHI2,IncHI2A,IncQ1	R	R	S	S	R	R	R	R	R	R	R	R	S
s13028	State Key Laboratory of Infectious Disease Prevention and Control	China	2012	feces	male	0.9	S83F/D87N	T57S/S80R		R	R	S	S	R	S	R	R	S	R	R	R	S
s13051	State Key Laboratory of Infectious Disease Prevention and Control	China	2012	feces	male	0.3	S83F/D87N	T57S/S80R	IncHI2,IncN,IncQ1,IncX1	R	R	S	S	R	R	R	R	S	S	R	R	S
s13069	State Key Laboratory of Infectious Disease Prevention and Control	China	2012	feces	female	0.5	S83F/D87G	T57S/S80R	IncA/C2,IncN	R	R	S	S	R	R	S	I	S	S	R	R	S
s14D10	State Key Laboratory of Infectious Disease Prevention and Control	China	2012	feces	female	74	S83F/D87N	T57S/S80R	ColpVC,IncA/C2,IncN	R	R	S	S	R	R	R	R	R	R	R	R	S
s14D102	State Key Laboratory of Infectious Disease Prevention and Control	China	2012	feces	female	0.6	S83F/D87N	T57S/S80R	IncX1	R	R	S	S	I	S	R	R	R	R	R	R	S
s15D023	State Key Laboratory of Infectious Disease Prevention and Control	China	2013	feces	male	9	S83F/D87N	T57S/S80R	IncHI2,IncHI2A,IncN,IncQ1	R	R	S	S	R	R	R	R	S	R	R	R	S
s15D030	State Key Laboratory of Infectious Disease Prevention and Control	China	2013	feces	male	56	S83F/D87N	T57S/S80R		R	R	S	S	I	R	S	R	S	R	R	R	S
s15D048	State Key Laboratory of Infectious Disease Prevention and Control	China	2013	feces	male	9m	S83F/D87G	T57S/S80R	IncA/C2,IncN	R	R	S	S	R	R	S	R	S	R	R	R	S
s15D050	State Key Laboratory of Infectious Disease Prevention and Control	China	2014	feces	male	0.6	S83F/D87N	T57S/S80R	IncA/C2	R	R	S	S	S	R	R	R	S	R	R	R	S
s15D058	State Key Laboratory of Infectious Disease Prevention and Control	China	2014	feces	female	56	S83F/D87G	T57S/S80R	IncHI2,IncHI2A	R	S	S	S	S	R	R	R	S	R	R	R	S
s15D077	State Key Laboratory of Infectious Disease Prevention and Control	China	2015	feces	female	1.1	S83F/D87N	T57S/S80R	IncHI2,IncHI2A,IncI1,IncN	R	R	S	S	S	S	R	R	S	S	R	R	S
s15D113	State Key Laboratory of Infectious Disease Prevention and Control	China	2015	feces	male	1.6	S83F/D87N	T57S/S80R		R	R	S	S	R	S	R	R	R	R	R	R	S
s15D135	State Key Laboratory of Infectious Disease Prevention and Control	China	2015	feces	male	2	S83F/D87N	T57S/S80R	IncR,IncX1	R	R	S	S	R	R	R	R	R	R	R	R	S
s16025	State Key Laboratory of Infectious Disease Prevention and Control	China	2015	feces	male	1.5	S83F/D87G	T57S/S80R	IncHI2,IncHI2A	R	R	S	S	R	S	R	R	R	R	R	R	S
s16054	State Key Laboratory of Infectious Disease Prevention and Control	China	2015	feces	male	22	S83F/D87N	T57S/S80R	IncHI2,IncHI2A,IncN	R	R	S	S	R	S	R	R	S	R	R	R	S
s16079	State Key Laboratory of Infectious Disease Prevention and Control	China	2015	feces	male	0.7	S83F/D87G	T57S/S80R	IncHI2,IncHI2A,IncN,IncQ1	R	R	S	S	R	R	R	R	S	R	R	R	S
s16096	State Key Laboratory of Infectious Disease Prevention and Control	China	2015	feces	female	1	S83F/D87N	T57S/S80R	IncHI2,IncHI2A,IncN	R	R	S	S	R	S	R	R	R	R	R	R	S
s16111	State Key Laboratory of Infectious Disease Prevention and Control	China	2015	feces	male	0.5	S83F/D87N	T57S/S80R		R	R	S	S	R	S	R	R	S	R	R	R	S
s17135	State Key Laboratory of Infectious Disease Prevention and Control	China	2016	feces	female	3.6	S83F/D87N	T57S/S80R		R	R	S	S	R	I	R	R	S	R	R	R	S
s17137	State Key Laboratory of Infectious Disease Prevention and Control	China	2016	feces	male	20	S83F/D87N	T57S/S80R	IncHI2,IncHI2A	R	R	S	S	R	R	R	R	R	R	R	R	S
s17140	State Key Laboratory of Infectious Disease Prevention and Control	China	2016	feces	male	0.7	S83F/D87N	T57S/S80R		R	R	S	S	R	S	R	R	S	R	R	R	S
s17141	State Key Laboratory of Infectious Disease Prevention and Control	China	2016	feces	male	0.4	S83F/D87N	T57S/S80R		R	R	S	S	R	S	R	R	S	R	R	R	S
s7029	State Key Laboratory of Infectious Disease Prevention and Control	China	2016	feces	male	0.9	S83F	T57S/S80R	IncHI2,IncHI2A,IncQ1	R	S	S	S	R	R	S	R	S	R	R	R	S
s7030	State Key Laboratory of Infectious Disease Prevention and Control	China	2017	feces	female	45	S83F	T57S/S80R	IncHI2,IncHI2A,IncN,IncQ1	R	S	S	S	S	R	S	R	S	R	R	R	S
s7031	State Key Laboratory of Infectious Disease Prevention and Control	China	2017	feces	male	38	S83F	T57S/S80R	IncHI2,IncHI2A,IncQ1	R	S	S	S	S	R	S	R	S	R	R	R	S
s7040	State Key Laboratory of Infectious Disease Prevention and Control	China	2017	feces	male	2.9	S83F	T57S/S80R	IncHI2,IncHI2A	R	S	S	S	R	R	S	R	R	R	R	R	S
s9079	State Key Laboratory of Infectious Disease Prevention and Control	China	2017	feces	female	2.3	S83F/D87G	T57S/S80R	IncHI2,IncHI2A	R	R	S	S	R	I	S	R	R	R	R	R	S
