# Supplementary material for: Dynamics of Antimicrobial Resistance and Genomic Epidemiology of Multidrug-Resistant Salmonella enterica Serovar Indiana ST17 from 2006 to 2017 in China
Source: mSystems. 2022 Jul 21;7(4):e00253-22. doi: 10.1128/msystems.00253-22 (PMC9426611; doi:10.1128/msystems.00253-22)
Supplement: TABLE S2 [file msystems.00253-22-s0002.docx]

**Table S2.**

| MIC value (mg/L) | No. of isolates | PMQR^a^ | | |  | QRDR (Amino acid substitution) | | | | | | | *p*-Value |
| --- | --- | --- | --- | --- | --- | --- | --- | --- | --- | --- | --- | --- | --- |
|  |  | One |  | Two |  | **ParC** | |  | **GyrA** | | | |  |
|  |  |  |  |  |  | T57S | T57S/S80R |  | S83F | D87G | S83F/D87G | S83F/D87N |  |
| ≤0.125 | 4 |  |  |  |  | 4(100%) |  |  |  | 4(100%) |  |  |  |
| 0.25 | 1 |  |  |  |  | 1(100%) |  |  |  | 1(100%) |  |  |  |
| 0.5 | 2 |  |  |  |  | 2(100%) |  |  | 2(100%) |  |  |  |  |
| 1 | 2 |  |  |  |  |  | 2(100%) |  | 2(100%) |  |  |  |  |
| 2 | 1 |  |  | 1(100%) |  | 1(100%) |  |  |  | 1(100%) |  |  |  |
| 4 | 2 | 1(50%) |  | 1(50%) |  | 1(50%) | 1(50%) |  | 1(50%) | 1(50%) |  |  |  |
| 8 | 24 | 2(8.3%) |  | 1(4.2%) |  |  | 24(100%) |  |  |  | 24(100%) |  |  |
| 16 | 51 | 9(16.7%) |  | 1(2.0%) |  |  | 51(100%) |  |  |  | 15(29.4%) | 36(70.6%) | 0.06093 |
| 32 | 58 | 39(67.2%) |  | 2(3.4%) |  |  | 58(100%) |  | 1(1.7%) |  | 33(56.9%) | 24(41.4%) | 0.00244 |
| 64 | 63 | 45(71.4%) |  | 16(25.4%) |  |  | 63(100%) |  |  |  | 16(25.4%) | 47(74.6%) | 0.0041 |
| 128 | 32 | 8(25.0%) |  | 24(75%) |  |  | 32(100%) |  |  |  | 9(28.1%) | 23(71.9%) | 0.12103 |
| 256 | 11 |  |  | 11(100%) |  |  | 11(100%) |  |  |  |  | 11(100%) | 0.00495 |
| Total | 251 | 104(41.4%) |  | 57(22.7%) |  | 9(3.6%) | 242(96.4%) |  | 6(2.4%) | 7(2.8%) | 97(38.6%) | 141(56.2%) |  |

a: PMQR, plasmid-mediated quinolone resistance

b: QRDRs, chromosomal quinolone resistance-determining regions
