# Supplementary material for: Dynamics of Antimicrobial Resistance and Genomic Epidemiology of Multidrug-Resistant Salmonella enterica Serovar Indiana ST17 from 2006 to 2017 in China
Source: mSystems. 2022 Jul 21;7(4):e00253-22. doi: 10.1128/msystems.00253-22 (PMC9426611; doi:10.1128/msystems.00253-22)
Supplement: TABLE S3 [file msystems.00253-22-s0003.docx]

**Table S3**.

| Antimicrobials classes | genes | No. of resistant isolates | | *p*-Value |
| --- | --- | --- | --- | --- |
|  |  | Human  isolates (n=120) | Food related isolates (n=131) |  |
| Aminoglycosides |  |  |  |  |
|  | *aac(3)-Ia* | 1(0.8%) | 0(0.0%) | 0.47809 |
|  | *aac(3)-IIa* | 2(1.7%) | 0(0.0%) | 0.22757 |
|  | *aac(3)-IId* | 7(5.8%) | 3(2.3%) | 0.20123 |
|  | *aac(3)-IVa* | 68(56.7%) | 58(44.3%) | 0.05828 |
|  | *aac(6')-Ib-cr* | 73(60.8%) | 73(55.7%) | 0.44365 |
|  | *aadA1* | 22(18.3%) | 9(6.9%) | <0.01** |
|  | *aadA15* | 2(1.7%) | 0(0.0%) | 0.22757 |
|  | *aadA17* | 1(0.8%) | 0(0.0%) | 0.47809 |
|  | *aadA2* | 9(7.5%) | 11(8.4%) | 0.82012 |
|  | *aadA22* | 1(0.8%) | 0(0.0%) | 0.47809 |
|  | *aadA5* | 52(43.3%) | 37(28.2%) | <0.05* |
|  | *aph(3')-Ia* | 37(30.8%) | 16(12.2%) | <0.01** |
|  | *aph(3')-IIa* | 17(14.2%) | 49(37.4%) | <0.01** |
|  | *aph(3')-VIa* | 0(0.0%) | 1(0.8%) | 1.0000 |
|  | *aph(4)-Ia* | 68(56.7%) | 58(44.3%) | 0.05828 |
|  | *armA* | 15(12.5%) | 22(16.8%) | 0.37621 |
|  | *rmtB* | 9(7.5%) | 10(7.6%) | 1.0000 |
|  | *aph(3'')-Ib* | 53(44.2%) | 41(30.6%) | <0.05* |
|  | *aph(6)-Id* | 52(43.3%) | 41(30.6%) | 0.05113 |
| Macrolides |  |  |  |  |
|  | *erm*(42) | 1(0.8%) | 0(0.0%) | 0.47809 |
|  | *erm*(B) | 1(0.8%) | 0(0.0%) | 0.47809 |
|  | *mph*(A) | 34(28.3%) | 50(38.2%) | 0.10932 |
|  | *mph*(E) | 4(3.3%) | 3(2.3%) | 0.71251 |
|  | *msr*(E) | 4(3.3%) | 3(2.3%) | 0.71251 |
| Sulphonamides |  |  |  |  |
|  | *sul1* | 69(57.5%) | 77(58.8%) | 0.89825 |
|  | *sul2* | 69(57.5%) | 66(50.4%) | 0.31071 |
|  | *sul3* | 20(16.7%) | 7(5.3%) | <0.01** |
| Trimethoprim |  |  |  |  |
|  | *dfrA1* | 2(1.7%) | 6(4.6%) | 0.28498 |
|  | *dfrA12* | 25(20.8%) | 18(13.7%) | 0.17938 |
| Fluoroquinolones |  |  |  |  |
|  | *qnrD* | 1(0.8%) | 0(0.0%) | 0.47809 |
|  | *qnrS1* | 0(0.0%) | 2(1.5%) | 0.49896 |
|  | *oqxAB* | 45(37.5%) | 22(16.4%) | <0.01** |
|  | *qepA* | 1(0.8%) | 0(0.0%) | 0.49896 |
|  | mutation(s) on *gyrA* | 120(100%) | 131(100%) | 1.0000 |
|  | mutation(s) on *parC* | 120(100%) | 131(100%) | 1.0000 |
| Rifamycin |  |  |  |  |
|  | *arr-2* | 0(0.0%) | 3(2.3%) | 0.248454 |
|  | *arr-3* | 74(61.7%) | 73(55.7%) | 0.37058 |
| Lincosamide |  |  |  |  |
|  | *lnu*(F) | 3(2.5%) | 0(0.0%) | 0.10784 |
| Fosfomycin |  |  |  |  |
|  | *fosA* | 42(35.0%) | 41(31.3%) | 0.59169 |
| Chloramphenicols |  |  |  |  |
|  | *catA1* | 17(14.2%) | 8(6.1%) | <0.05* |
|  | *catB3* | 74(61.7%) | 73(55.7%) | 0.37058 |
|  | *cmlA1* | 22(18.3%) | 11(8.4%) | <0.05* |
|  | *floR* | 76(63.3%) | 68(51.9%) | 0.07457 |
| Penicillins |  |  |  |  |
|  | *bla*_OXA-1_ | 73(60.8%) | 73(55.7%) | 0.44365 |
|  | *bla*_OXA-10_ | 0(0.0%) | 3(2.3%) | 0.24845 |
|  | *bla*_TEM-1B_ | 31(25.8%) | 34(26.0%) | 1.0000 |
|  | *bla*_TEM-209_ | 0(0.0%) | 4(3.1%) | 0.12345 |
| Tetracyclines |  |  |  |  |
|  | *tet*(A) | 84(70.0%) | 77(58.8%) | 0.06692 |
|  | *tet*(B) | 0(0.0%) | 1(0.8%) | 1.0000 |
|  | *tet*(M) | 9(7.5%) | 1(0.8%) | <0.01** |
|  | *tet*(Q) | 0(0.0%) | 1(0.8%) | 1.0000 |
| Third-generation  cephalosporins |  |  |  |  |
|  | *bla*_CMY-2_ | 0(0.0%) | 9(6.9%) | <0.01** |
|  | *bla*_CTX-M-123_ | 1(0.8%) | 0(0.0%) | 0.47809 |
|  | *bla*_CTX-M-14_ | 19(15.8%) | 14(10.7%) | 0.26412 |
|  | *bla*_CTX-M-15_ | 9(7.5%) | 0(0.0%) | <0.01** |
|  | *bla*_CTX-M-27_ | 4(3.3%) | 1(0.8%) | 0.19192 |
|  | *bla*_CTX-M-55_ | 15(12.5%) | 15(11.5%) | 0.84712 |
|  | *bla*_CTX-M-65_ | 28(23.3%) | 32(24.4%) | 0.88287 |
