# Supplementary figures and images for: Dynamics of Antimicrobial Resistance and Genomic Epidemiology of Multidrug-Resistant Salmonella enterica Serovar Indiana ST17 from 2006 to 2017 in China
Source: mSystems. 2022 Jul 21;7(4):e00253-22. doi: 10.1128/msystems.00253-22 (PMC9426611; doi:10.1128/msystems.00253-22)

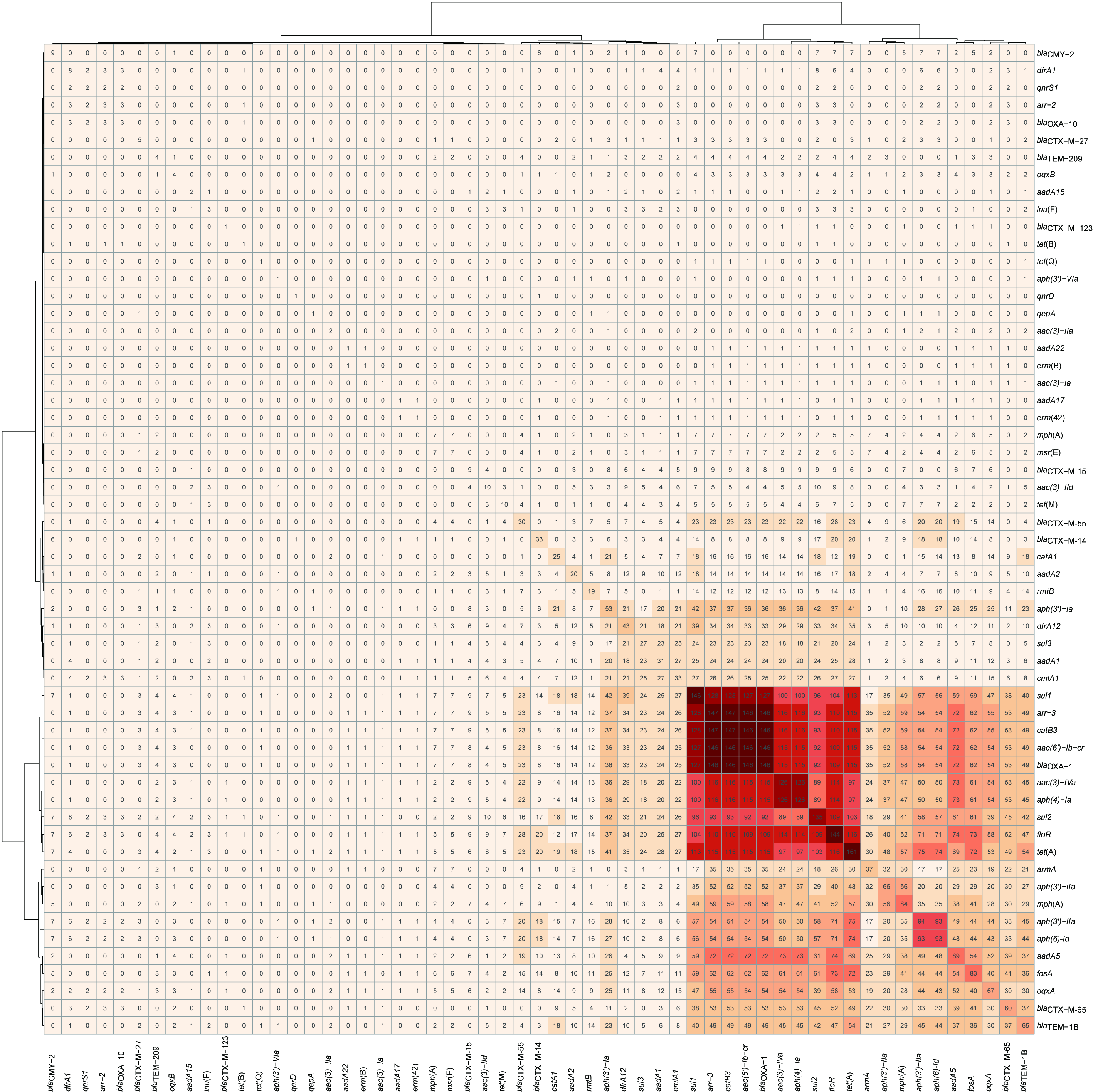

Supplement: FIG S1 [file msystems.00253-22-s0004.tif]

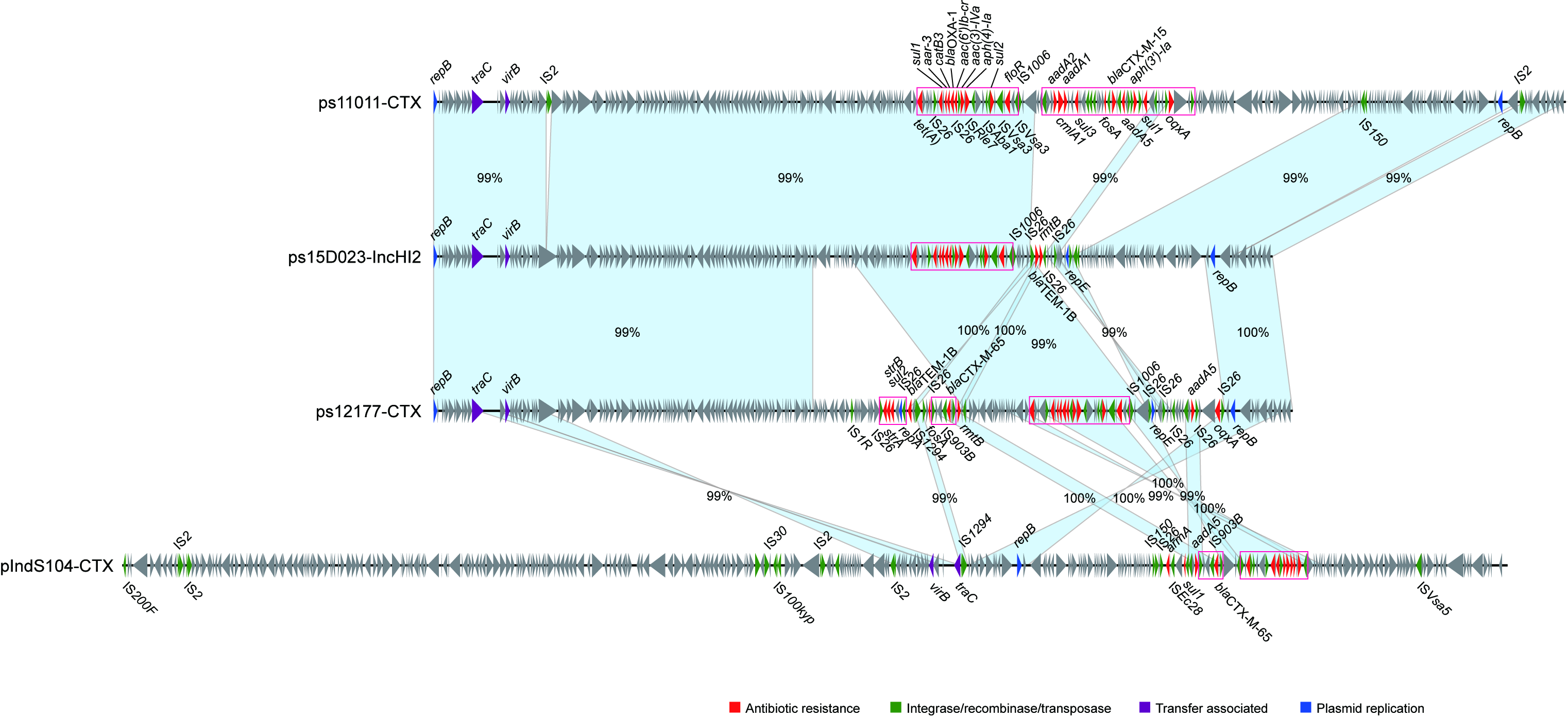

Supplement: FIG S2 [file msystems.00253-22-s0005.tif]

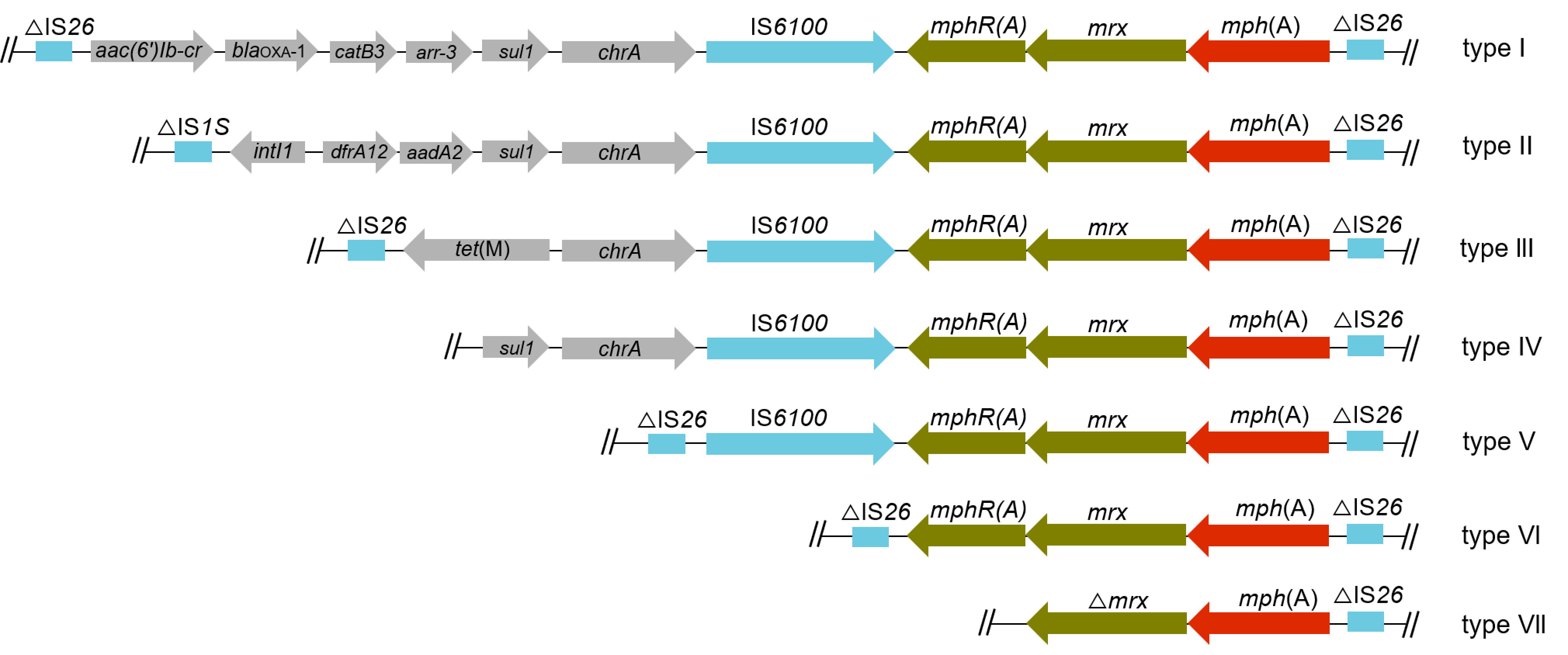

Supplement: FIG S3 [file msystems.00253-22-s0006.tif]
